# Relation equivariant graph neural networks to explore the mosaic-like tissue architecture of kidney diseases on spatially resolved transcriptomics

**DOI:** 10.1093/bioinformatics/btaf303

**Published:** 2025-05-13

**Authors:** Mauminah Raina, Hao Cheng, Ricardo Melo Ferreira, Treyden Stansfield, Chandrima Modak, Ying-Hua Cheng, Hari Naga Sai Kiran Suryadevara, Dong Xu, Michael T Eadon, Qin Ma, Juexin Wang

**Affiliations:** Department of Biomedical Engineering and Informatics, Indiana University Indianapolis, Indianapolis, IN 46202, United States; Department of Biomedical Informatics, College of Medicine, The Ohio State University, Columbus, OH 43210, United States; Department of Medicine, Indiana University, Indianapolis, IN 46202, United States; Department of Biomedical Engineering and Informatics, Indiana University Indianapolis, Indianapolis, IN 46202, United States; Department of Biomedical Engineering and Informatics, Indiana University Indianapolis, Indianapolis, IN 46202, United States; Department of Medicine, Indiana University, Indianapolis, IN 46202, United States; Department of Biomedical Engineering and Informatics, Indiana University Indianapolis, Indianapolis, IN 46202, United States; Department of Electrical Engineering and Computer Science, and Christopher S. Bond Life Sciences Center, University of Missouri, Columbia, MO 65211, United States; Department of Medicine, Indiana University, Indianapolis, IN 46202, United States; Department of Biomedical Informatics, College of Medicine, The Ohio State University, Columbus, OH 43210, United States; Department of Biomedical Engineering and Informatics, Indiana University Indianapolis, Indianapolis, IN 46202, United States

## Abstract

**Motivation:**

Chronic kidney disease (CKD) and acute kidney injury (AKI) are prominent public health concerns affecting more than 15% of the global population. The ongoing development of spatially resolved transcriptomics (SRT) technologies presents a promising approach for discovering the spatial distribution patterns of gene expression within diseased tissues. However, existing computational tools are predominantly calibrated and designed on the ribbon-like structure of the brain cortex, presenting considerable computational obstacles in discerning highly heterogeneous mosaic-like tissue architectures in the kidney. Consequently, timely and cost-effective acquisition of annotation and interpretation in the kidney remains a challenge in exploring the cellular and morphological changes within renal tubules and their interstitial niches.

**Results:**

We present an empowered graph deep learning framework, REGNN (Relation Equivariant Graph Neural Networks), designed for SRT data analyses on heterogeneous tissue structures. To increase expressive power in the SRT lattice using graph modeling, REGNN integrates equivariance to handle n-dimensional symmetries of the spatial area, while additionally leveraging Positional Encoding to strengthen relative spatial relations of the nodes uniformly distributed in the lattice. Given the limited availability of well-labeled spatial data, this framework implements both graph autoencoder and graph self-supervised learning strategies. On heterogeneous samples from different kidney conditions, REGNN outperforms existing computational tools in identifying tissue architectures within the 10× Visium platform. This framework offers a powerful graph deep learning tool for investigating tissues within highly heterogeneous expression patterns and paves the way to pinpoint underlying pathological mechanisms that contribute to the progression of complex diseases.

**Availability and implementation:**

REGNN is publicly available at https://github.com/Mraina99/REGNN.

## 1 Introduction

The kidneys play several vital roles in maintaining bodily equilibrium, including filtrating bodily fluids and waste, regulating blood acid-base balance, maintaining electrolyte balance, and supporting the production of red blood cells (Murray and Paolini 2024). Chronic kidney disease (CKD) and acute kidney injury (AKI) are two of the most common diseases worldwide. CKD has a prevalence of approximately 13.4%, with 5–7 million patients experiencing kidney failure in late-stage CKD (Johansen *et al.* 2021), and AKI was found to have a prevalence of up to 3000 cases in 1 million hospitalized patients (Safari *et al.* 2018). Even acute and chronic cellular and morphological changes occur in renal tubules surrounding the interstitial niche (Ferreira *et al.* 2021), there are still many unknowns in understanding the biological and pathological mechanisms of CKD and AKI, especially how various cells play different roles in key injury-related processes such as fibrosis (Kuppe *et al.* 2021), immune infiltration (Allison 2019), and epithelial repair (Ferreira *et al.* 2021) in different kidney tissues.

The emergence of spatially resolved transcriptomics (SRT) has brought novel advancements and opportunities in uncovering the fundamental pathogenesis behind a wide range of human diseases (Moses and Pachter 2022). The increasing availability of SRT data (Xiaowei 2021) is enabling novel analysis to reshape our understanding of cell spatial organization and their functional generation (Rao *et al.* 2021), including cell–cell communications (Jin *et al.* 2021), spatially variable genes relating to spatial development (Svensson *et al.* 2018, Wang *et al.* 2023), and analysis of tissue architecture (Elosua-Bayes *et al.* 2021). These insights would be vital in interpreting the underlying biological and pathological processes in kidney tissues involved in CKD and AKI (Gisch *et al.* 2024).

**Figure 1.**
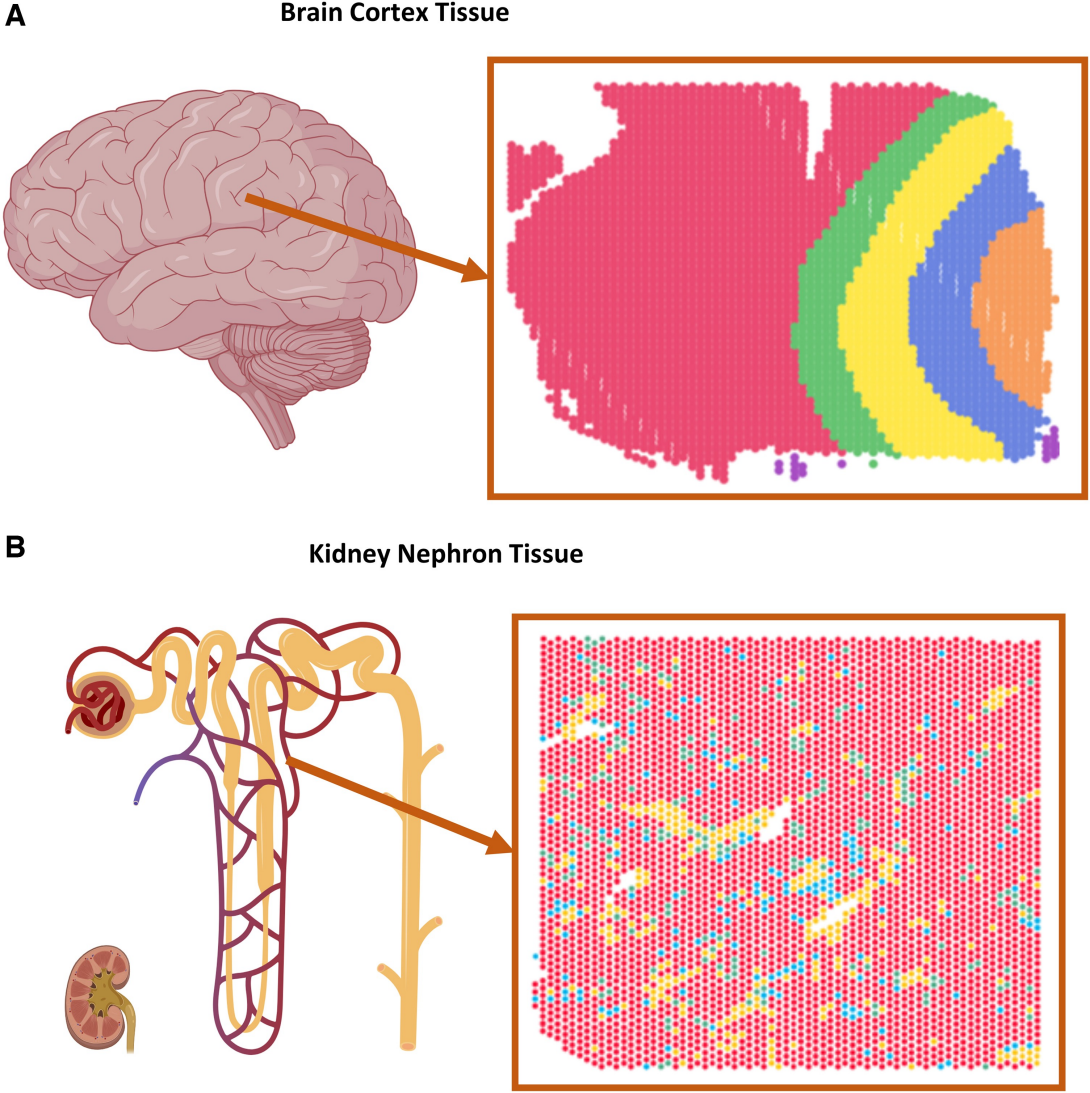
Comparison of the tissue architecture and cell type distribution of the brain cortex vs kidney nephron. (A) Brain cortex diagram and ribbon-like cell types distribution of a brain cortex sample (Maynard *et al*. 2021) on 10× Visium platform. (B) Kidney nephron diagram and mosaic-like cell type distribution of a kidney sample from KPMP (de Boer *et al*. 2021) on 10× Visium platform. Each color represents a different cell type present in the tissue. For Fig 1A-B, the Brain and Kidney drawing was sourced from BioRender.

Analyzing SRT data on kidney diseases presents three key challenges: First, modeling the kidney’s heterogeneous, sparse, and mosaic-like cell types differs from organs with ribbon-distributed regions, as kidney cells exist in close proximity with varied distribution across pathologic sections (Lake *et al.* 2023). In a pathologic section, kidney tubules are cut from different directions during the sample preparation, which causes more cell-type distribution variation across samples. This complexity poses a huge challenge in methods development that are often trained and benchmarked on brain tissue structures. Figure 1 illustrates these tissue type distinctions on 10× Visium SRT (Ferreira *et al.* 2021, Zhao *et al.* 2021). The second challenge is the limited expressive power of graph neural networks. Classical message passing GNN(Kipf and Welling 2016) is theoretically limited by the 1-order Weisfeiler-Lehman (1-WL) test (Xu *et al.* 2018) to distinguish if two given graphs are isomorphic or not (Balcilar *et al.* 2021). Moreover, the nature of the regular topology in the lattice structures of the SRT makes it difficult to differentiate between nodes for they have similar topology in the modeled lattice graphs. This graph symmetry, such as the hexagonal lattice in 10× Visium spots, can obscure relationships between neighboring nodes, especially with sparse kidney cell-type distributions. Third, efficiently annotating vast omics datasets remains challenging, as even with AI-based tools, linking histology to SRT spots can require weeks of pathologist time (Elmarakeby *et al.* 2021, Quan *et al.* 2023, Shaban *et al.* 2024), making the process expensive and sometimes unfeasible.

Currently, numerous computational methods are being developed to identify tissue architecture from SRT data. BayesSpace (Zhao *et al.* 2021) uses Bayes inference to dissolve tissue architecture, and Giotto (Dries *et al.* 2021) utilizes graph-based clustering methods for spatial clustering. For cell-type clustering, FICT (Teng *et al.* 2022) combines expression and neighborhood for assignment, while smFISHhmrf (Zhu *et al.* 2018) uses a Hidden–Markov random field approach to find neighborhood patterns among cells. Graph neural networks (GNNs) (Juexin Wang *et al.* 2021) model cell relations as a graph on SRT and learn low dimensional representations through deep learning architectures. Both SpaGCN (Hu *et al.* 2021) and CCST (Li *et al.* 2022) use deep learning to categorize the spatial domain based on graph neural networks (Ferreira *et al.* 2021). Methodologies such as SEDR (H. Xu *et al.* 2024) and STAGATE (Dong and Zhang 2022) build on graph autoencoder strategies for learning their low-dimensional latent embeddings. RESEPT (Chang *et al.* 2022) combines GNNs with the RestNet50 model to process image segmentation as cell types (He *et al.* 2016). SpaGCN (Hu *et al.* 2021), SiGra (Tang *et al.* 2023), and GraphST (Long *et al.* 2023) additionally adopt H&E image data to improve the model performances. Despite these advances, significant gaps remain in robustly analyzing heterogeneous tissue structures, particularly in kidney studies.

To address these challenges, we introduce an empowered graph deep learning framework, REGNN (Relation Equivariant Graph Neural Networks), for SRT data analyses on heterogeneous tissue structures. To increase the expressive power in the SRT lattice using graph modeling, the proposed REGNN integrates three strategies to address current challenges:

1. Equivariance to handle the rotational and translational symmetries of the spatial space.
2. Positional encoding (PE) to identify and strengthen the relative spatial relations of the nodes uniformly distributed in the lattice.
3. A graph self-supervised learning (SSL) strategy (Yang et al. 2020) to generate robust data representations.

The key advantage of including equivariance and positional encoding lies in the ability to capture and leverage the inherent spatial relationships and symmetries present in SRT data. Equivariance allows the model to handle rotational and translational symmetries in the given spatial domain, and this ensures that the analysis remains consistent regardless of the orientation or position of the tissue sample (H. Chen *et al.* 2024). This is particularly important in biological contexts such as the kidney, where the relative arrangement of cells and their interactions are crucial, but the absolute orientation may vary between samples. Implementing positional encoding components further strengthens the model’s capability by allowing it to identify and leverage the relative spatial relationships between nodes distributed across the SRT tissue sample (Wang *et al.* 2022). These features, combined with a graph self-supervised learning strategy, empower the model to generate robust data representations that accurately reflect the underlying biological structures and processes (Liu *et al.* 2024). This empowered GNN design would ideally lead to more accurate and biologically meaningful insights from spatial transcriptomics data, potentially revealing new patterns of gene expression and cellular organization that are critical for understanding tissue function and disease processes.

The combination of these techniques empowers REGNN over classical graph neural networks (GNN) and makes it capable of tackling challenging heterogeneous mosaic-like kidney samples. REGNN achieves state-of-the-art performance on 23 mosaic-like kidney samples of 10× Visium SRT data from the KPMP (Kidney Precision Medicine Project) atlas (de Boer *et al.* 2021). We also demonstrate the performance of REGNN correlates with tissue heterogeneity, which shows its potential in other highly heterogeneous tissues such as the kidney.

## 2 Materials and methods

**Figure 2.**
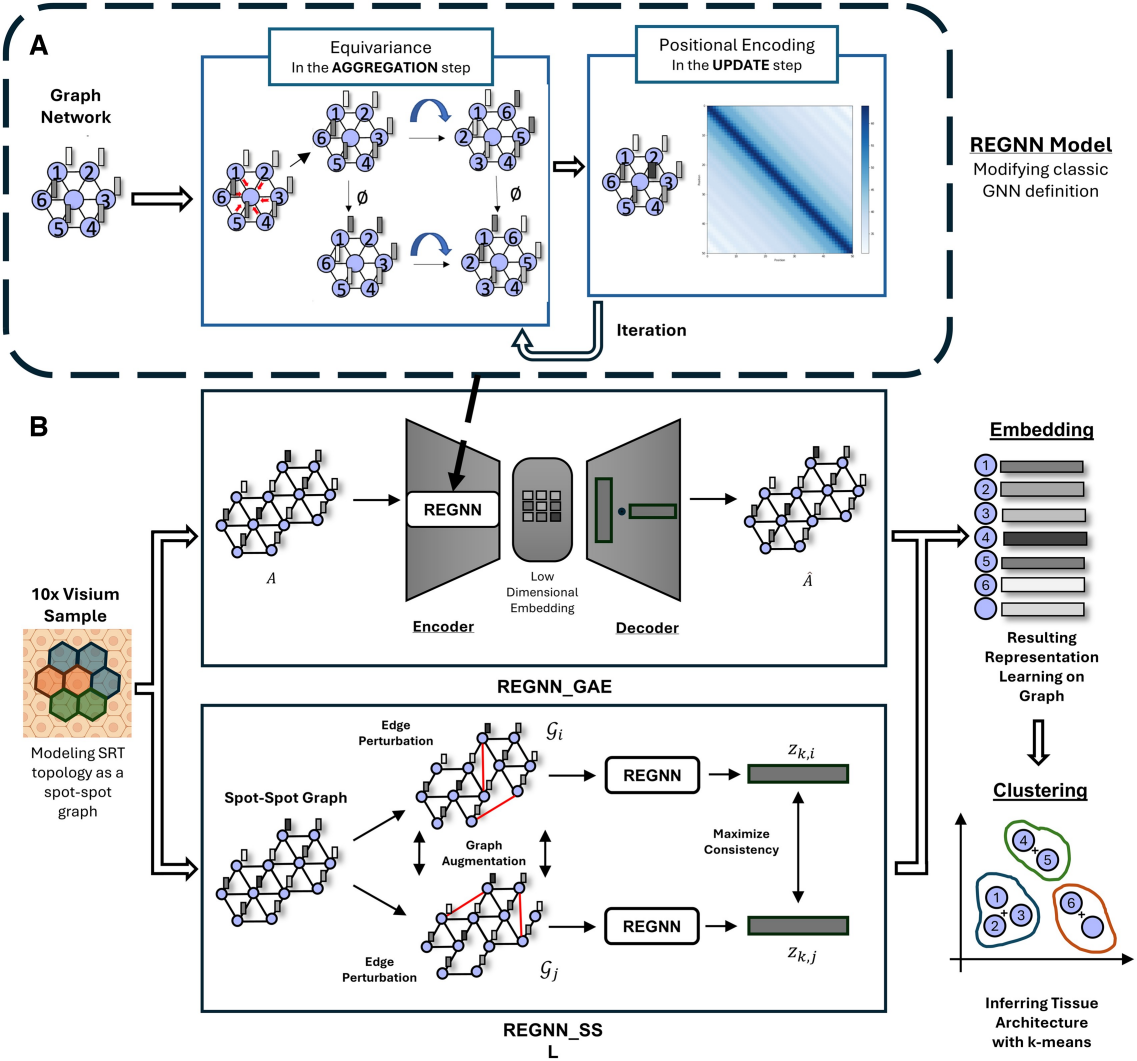
Schema of REGNN model. Take 10× Visium platform as an example, REGNN models the SRT data as a spot-spot graph and learns the embeddings of the data, then infers tissue architecture through clustering. (A) The empowered REGNN contains: (1) Equivariance in AGG operation. (2) Positional Encoding in UPDATE operation. (B) REGNN_GAE as a graph autoencoder architecture built on REGNN, and (C) REGNN_SSL as a graph contrastive learning strategy built on REGNN.

REGNN is an empowered graph neural network identifying heterogeneous tissue structures in SRT data, which is designed to capture the comprehensive relations between the spots in SRT by keeping their spatial relations equivariant and unique in the learnt representation. Building on the classical message passing GNN, REGNN incorporates two critical components to increase expressive power, namely, equivariance and PE. REGNN can be trained either in an unsupervised learning style graph autoencoder (GAE) as REGNN_GAE or a self-supervised learning strategy REGNN_SSL. This framework learns low dimensional presentations of each spot in the SRT and infers tissue architectures through clustering the embeddings. The schema of the proposed framework is shown in Fig. 2A.

### 2.1 Graph modeling SRT and graph neural networks

Based on our previous works RESEPT (Chang *et al.* 2022) and scGNN (Juexin Wang *et al.* 2021), SRT data are represented as a spatial spot-spot graph G={A,X} by defining adjacency matrix A, $A\in R^{\left|V|\times |V\right|}$ with node attributes X, $X\in R^{|V|\times D}$, where V is the node set with D dimensions from gene expression. Each spot within a tissue sample containing several single cells is modeled as a node v. In graph G, measured gene expression values of the spot are treated as the node attributes X, and the neighboring spots directly adjacent in the Euclidean space on the tissue slice are linked with an undirected edge e. As a result, this modeled undirected graph represents both the spatial context and the expression similarities between SRT nodes. This graph modeling method is applicable for both 10× Visium and FISH platforms.

Generally, a classical L-layer graph convolution network (GCN) includes two steps of operations for each node $v_{i}$ at each layer: (i) AGG operation: aggregating messages $m_{i}^{l}$ from the neighborhood $N_{i}$ at lth layer as Equation (1); (ii) UPDATE operation: updating node representation with $h_{i}^{l}$ as Equation (2). The representation of each node $h_{i}^{l+1}$ at layer l+1 can be learned from the AGG and UPDATE operations.


(1) $$m_{i}^{l}=\mathrm{AGG}(\left\{h_{j}^{l}:v_{j}{\in N}_{i}\right\})$$

(2) $$h_{i}^{l+1}=\mathrm{UPDATE}(h_{i}^{l},m_{i}^{l})$$

### 2.2 REGNN incorporates equivariance in AGG operation

For SRT in ST and 10×X Visium technologies, directly modeling its lattice structure as a spot graph brings rotational and translational symmetries with the spatial gene expression pattern, which confuses and diminishes the expressive power of classical GNNs. Where E(3) GNNs only maintain transformations within the 3D space, E(n)-equivariant approaches remain consistent under rigid transformations—such as translation, rotation, reflection, and permutation—in any n-dimensional space. Inspired by the superior scalability of E(n) equivariant GNN (Satorras *et al.* 2021), REGNN integrates translation, rotation, reflection, and permutation equivariance with respect to an input set of spots in the modelled spatial spot–spot graph in the AGG operation. Targeting lattice symmetries, we define coordinates embedding x for each spot, where $x\in R^{|V|\times 2}$ in 2D SRT data. The initial $x^{0}$ is the actual X–Y coordinates in the SRT lattice. Equivariance is integrated by modifying the classic GCN layer’s definition to include the learning of coordinates embeddings associated with each graph node(Sato 2020). For connected node i and j in the spot-spot graph at the lth layer, REGNN defines message embedding $m_{ij}$ in Equation (3) to incorporate the relative square distance between coordinates ${\left|\left|x_{i}^{l}-x_{j}^{l}\right|\right|}^{2}$, this information together with node embeddings $h_{i}^{l},\;h_{j}^{l},\;$and edge attributes $e_{ij}$ are summarized by learnable Multilayer Perceptrons (MLPs)${\;\varphi }_{e}$. Then coordinate embedding $x_{i}^{l+1}$ is updated by a weighted sum of all relative differences between coordinates with $m_{ij}$ in Equation (4), where${\;\varphi }_{x}$ is another MLPs, C is a tunable hyperparameter to control the speed and strength. Compared with the classical AGG operation in Equation (1), REGNN updates the AGG operation by aggregating messages from all edges in Equation (5).


(3) $$m_{ij}={\;\varphi }_{e}\;(h_{i}^{l},\;h_{j}^{l},\;{\left|\left|x_{i}^{l}-x_{j}^{l}\right|\right|}^{2},\;e_{ij})$$

(4) $$x_{i}^{l+1}=x_{i}^{l}+C\sum_{j\neq i}(x_{i}^{l}-x_{j}^{l}){\;\varphi }_{x}(m_{ij})$$

(5) $$m_{i}^{l}=\mathrm{AGG}(\left\{m_{ij}|i\neq j\right\})=\sum_{j\neq i}m_{ij}$$

### 2.3 REGNN incorporates positional encoding in UPDATE operation

Besides integrating equivariance in handling symmetries in AGG operation, REGNN also incorporates PE in the following-up UPDATE operation. Widely used in linear data structures such as Transformers (Vaswani 2017) and large language models (Touvron *et al.* 2023), PE is often treated as a unique feature to mark the entity’s relative position. In the SRT modeling, PE shows and strengthens the spatial relations between the nodes in the graph. Moreover, PE can be applied to the model to increase its discerning power to distinguish on an isomorphic graph. We use one widely accepted strategy in PE, utilizing sinusoids in the 2D space (Vaswani 2017: 5998–6008, Zhong *et al.* 2021). Thus, each coordinate is featured with a fixed PE consisting of *E* sinusoids with wavelengths that follow a geometric progression from 1 to the Nyquist limit, where E∈[1,|V|] is the size of the SRT and k represents the coordinates of the SRT:


(6) $$\begin{array}{c} {PE}^{\left(2i\right)}(k_{j})=\sin\left(k_{j}E\pi {\left(\frac{2}{E}\right)}^{\frac{i}{\frac{2}{E}-1}}\right),\;i=0,\ldots ,\frac{2}{E}-1;k_{j}\in k\\ {\;PE}^{\left(2i+1\right)}(k_{j})=\cos\left(k_{j}E\pi {\left(\frac{2}{E}\right)}^{\frac{i}{\frac{2}{E}-1}}\right),\;i=0,\ldots ,\frac{2}{E}-1;k_{j}\in k \end{array}$$

In REGNN, UPDATE operation as Equation (2) in classical GNN is adjusted by explicitly adding the PE embedding to message embedding $m_{i}^{l}$ as operation ⊕ in Equation (7), where α is the intensity of PE.


(7) $$h_{i}^{l+1}=\mathrm{UPDATE}(h_{i}^{l},m_{i}^{l}\;⊕\;\alpha *{PE}_{i})$$

Compared to classical L-layer GCN, REGNN uses the idea of equivariance in AGG operation as Equation (5) and integrates PE in UPDATE operation as Equation (7), the final formulation of REGNN is shown as Equation (8).


(8) $$h_{i}^{l+1}=\mathrm{UPDATE}(h_{i}^{l},\mathrm{AGG}(\left\{m_{ij}|i\neq j\right\})\;⊕\;\alpha *{PE}_{i})$$

### 2.4 REGNN_GAE learns node embeddings using a graph autoencoder in an unsupervised learning strategy

REGNN can be trained in an unsupervised learning strategy where it is adopted as the backbone architecture in REGNN_GAE framework as Fig. 2B. The proposed REGNN is utilized as the encoder of the graph autoencoder, and graph embedding Z is learnt by stacking two layers of REGNN in Equation (9). The decoder calculates the inner product of Z, and then activated by sigmoid activation function to reconstruct adjacency matrix$\;\hat{A}$ in Equation (10).


(9) Z=REGNN(REGNN(X,A,x, e), A,x, e)

(10) $$\hat{A}=\mathrm{sigmoid}(Z,\;Z^{T})$$

The loss function of the graph autoencoder is minimizing cross-entropy L between reconstructed matrix $\hat{A}$ and input adjacency matrix A as shown in Equation (11). With N nodes being the number of spots on the slide sample, N×N is the dimension of the adjacency matrix, and $a_{ij}\;\mathrm{and}\;{\hat{a}}_{ij}$ are the elements of A and $\hat{A}$ respectively.


(11) $$L(A,\hat{A})=-\;\frac{1}{N\times N}\sum_{i=1}^{N}\sum_{j=1}^{N}\left(a_{ij}*\log\left({\hat{a}}_{ij}\right)+(1-a_{ij})*\log(1-{\hat{a}}_{ij})\right)$$

### 2.5 REGNN_SSL implements self-supervised learning through graph contrastive learning

Inspired by GraphCL (You *et al.* 2020), we adopted a graph based contrastive learning strategy to learn the intrinsic node-wise low-dimensional representation in REGNN_SSL (Fig. 2B). The primary objective of REGNN_SSL is to enhance mutual information between representations of two types of augmented graphs derived from the input spot graph. Firstly, the input SRT spot graph G is augmented with data augmentation strategies at the graph level. These graph augmentation strategies generate many new augmented graphs G by randomly perturbate the original topology of the input graph at different levels of node, edge, attribute, and subgraph with specific ratio r, 0<r<1. During GNN pre-training, SSL is optimized to contrastive loss of N pairs of randomly sampled augmented graphs. For kth pair of augmented graphs $G_{i}$ and $G_{j}$, REGNN learnt the corresponding graph level embedding $z_{k,i}$ and $z_{k,j}$. Negative pairs are generated from the other N−1 augmented graphs using the same strategy as simCLR (T. Chen *et al.* 2020). The contrastive loss function is defined to maximize the consistency between positive pairs compared with the negative pairs with normalized temperature-scaled cross entropy loss (Sohn 2016), which summarizes all positive pairs as Equation (12).


(12) $$\mathrm{Loss}=\sum_{k=1}^{N}-\log\frac{\exp\left(\frac{\mathrm{sim}(z_{k,i},z_{k,j})}{\tau }\right)}{\sum_{k^{\prime }=1,k^{\prime }\neq k}^{N}\exp\left(\frac{\mathrm{sim}(z_{k,i},z_{k^{\prime },j})}{\tau }\right)}$$

where $\mathrm{sim}(z_{k,i},z_{k,j})=z_{k,i}^{T}z_{k,j}/\|z_{k,i}\|\|z_{k,j}\|$, which is defined as the cosine similarity function, τ denotes the temperature parameter.

We assume the learnt graph embedding Z from both REGNN_GAE and REGNN_SSL represent the topological relations within the graph. Subsequently, we apply the k-means clustering algorithm on Z, and then annotate the clustering results as the known distinct cell types within the tissue architecture.

### 2.6 Data processing

All SRT sample data was extracted using a similar preprocessing procedure. For both 10x Visium and ST data, gene expression counts, spatial coordinates, and cell type labels were extracted using R and then stored in separate input files. These counts, coordinates, and label files were the inputs used to test each method. Any further preprocessing would follow the recommended steps of the tested method, as described in their official documentation (Supplementary Notes 2). The following subsections cover the samples used to test REGNN.

#### 2.6.1 Kidney data

Twenty-three kidney samples with 10× genomics Visium platform are processed and provided by the KPMP at https://atlas.kpmp.org/repository/. The counts matrix, spatial data, and metadata annotation labels are extracted using Seurat.

#### 2.6.2 SpatialLIBD

The spatialLIBD (Pardo *et al.* 2022) dataset consists of portions of the human dorsolateral prefrontal cortex (DLPFC) within 12 samples with 10× genomics Visium platform. This processed data included the LogCPM and SCTransform processed counts along with the spot annotations of each sample can be found at https://research.libd.org/spatialLIBD/.

#### 2.6.3 Human breast cancer

One human breast cancer sample is available through 10x genomics website at https://www.10xgenomics.com/datasets/human-breast-cancer-block-a-section-1-1-standard-1-0-0. The preprocessed counts and annotated spot data is publicly available through SEDR’s (H. Xu *et al.* 2024) GitHub repository.

#### 2.6.4 HER2-positive breast tumor

There are eight samples on the ST platform in the collection of HER2-positive breast tumor with manual annotation at https://github.com/almaan/her2st. This data included the preprocessed counts along with the spatial spot coordinates and pixel coordinates.

### 2.7 Functional tissue unit domain annotation

Within kidney samples, glomeruli and the tubulointerstitium are annotated visually using the hematoxylin and eosin stain in Loupe Browser 6.0. A spot is denoted glomerular if its centroid falls within the bounds of Bowman’s capsule. Spots found on the edge of tissue are eliminated from the analysis to prevent edge artifacts. All other spots are considered tubulointerstitial. All the cell types are manually validated by experienced nephrology physicians from KPMP.

## 3 Results

### 3.1 REGNN accurately identifies the tissue architecture of mosaic-like heterogeneous kidney samples

Twenty-three kidney samples from healthy, CKD, and AKI patients sequenced on 10× Visium platform are downloaded from the KPMP atlas (de Boer *et al.* 2021). The sample information is detailed in Supplementary Table 1. Cell types identified in the spots of the samples are annotated as epithelial, endothelial, immune, and stromal by experienced nephrology physicians from KPMP. These annotations are utilized as the gold standard benchmarks to test the performance of REGNN and other existing methods including BayesSpace (Zhao *et al.* 2021), Giotto (Dries *et al.* 2021), SpaGCN (Hu *et al.* 2021), RESEPT (Chang *et al.* 2022), SiGra (Tang *et al.* 2023), SEDR (H. Xu *et al.* 2024), GraphST (Long *et al.* 2023), and STAGATE (Dong and Zhang 2022), CCST (Li *et al.* 2022), FICT (Teng *et al.* 2022), and smFISHhmrf (Dries *et al.* 2021). Both versions of REGNN are then tested separately to observe the difference in performance between the GAE and SSL frameworks. Four criteria were used to quantify the efficacy of these SRT analysis tools, including Adjusted Rand Index (ARI), Rand Index (RI), Normalized Mutual Info score (NMI), and Fowlkes Mallows Index (FMI) (Rodriguez *et al.* 2019).

**Figure 3.**
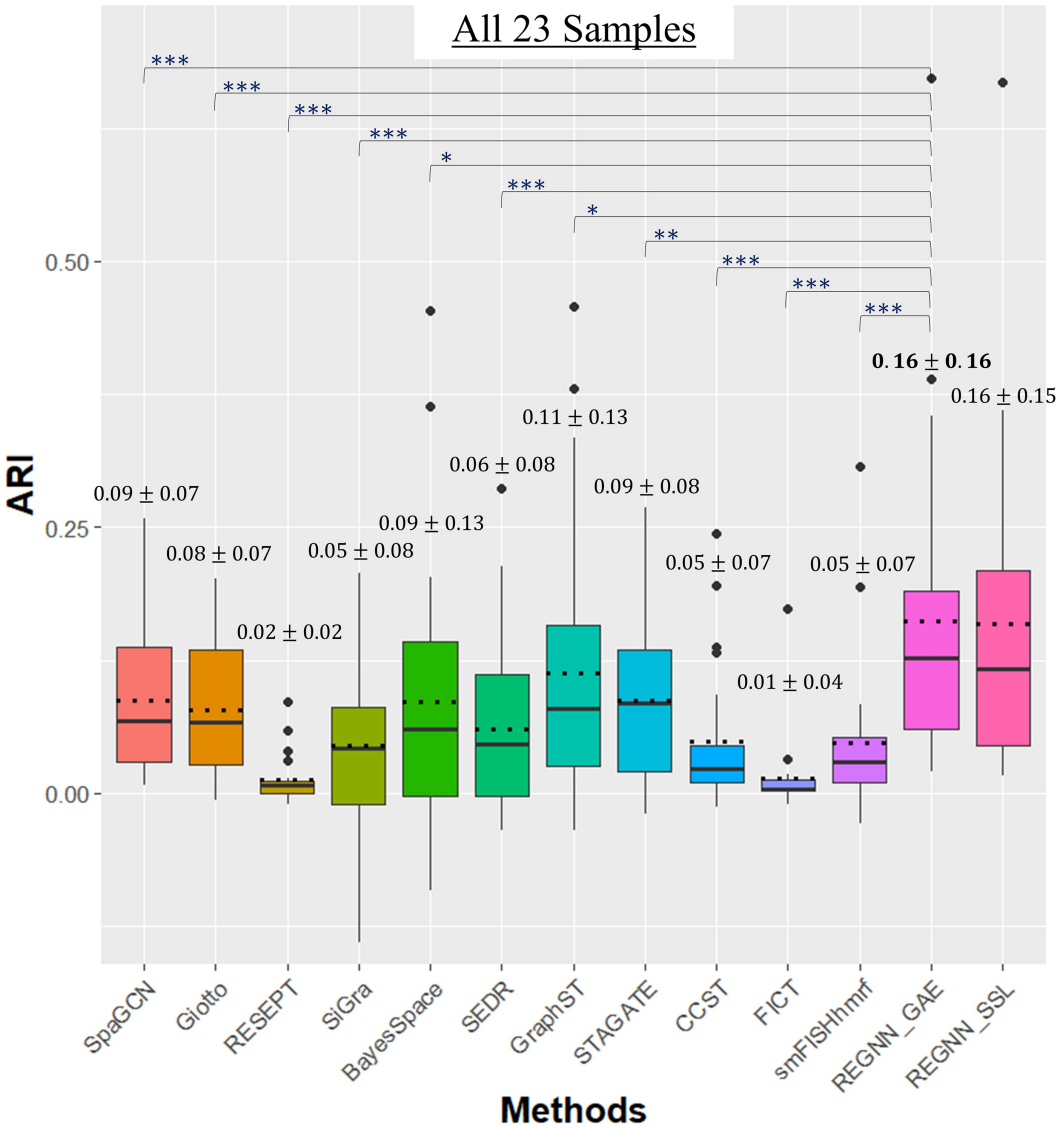
Performance comparison on ARI in all 23 samples from KPMP. For each method, the median is shown by the solid black line and the median is displayed by the dotted line. The Wilcoxon signed rank test is performed to determine the significance of REGNN_GAE’s mean compared to other competitive methods. REGNN_GAE and REGNN_SSL have no significant difference between their result means.

For all 23 samples, we observe that both REGNN-based frameworks outperform the competitive methods within a larger median and significantly larger means within the ARI performance as seen in Supplementary Table S7. The box plot of all samples is shown in Fig. 3 and Supplementary Fig. S1. Additionally, it is observed that REGNN_SSL has a slightly smaller standard deviation when compared to REGNN_GAE, though REGNN_GAE is able to achieve a higher performance. Specifically, we can observe that both REGNN frameworks achieved better or comparable performance in ARI among 11 CKD samples, while the healthy reference samples appear to cause some trouble for the model (Supplementary Figs 2–4). Besides the best performer, REGNN, we can see that GNN-based methods, including SpaGCN, SiGra, GraphST, and STARGATE, generally outperform non-deep learning-based approaches in these samples. GraphST, which integrates H&E images and adopts a self-supervised learning strategy, demonstrates second-best performance in all samples, illustrating the potential of both self-supervised learning and image integration. Considering the performance of the other two methods integrating image information, SpaGCN and SiGra, the image integration strategy needs more careful design and implementation. Similarly, the Bayesian method BayesSpace achieves good results in some cases, but there is high variance in all the samples.

**Figure 4.**
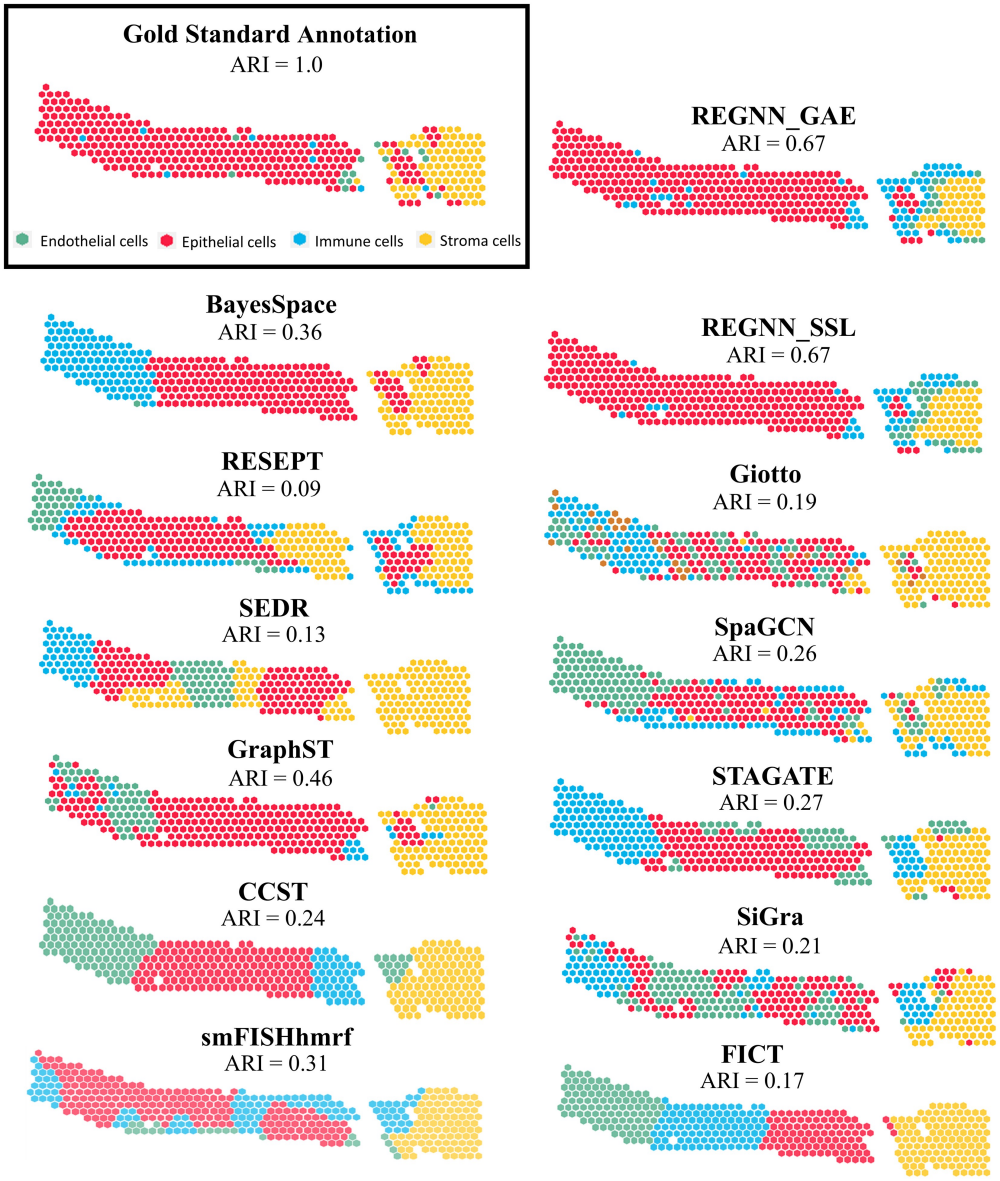
Visualization of results from computational methods on a representative CKD sample. The gold standard annotations and calculated results of the computational methods are mapped to the original locations of CKD sample V10S14-085_XY04_21-0057.

A sample V10S14-085_XY04_21–0057 with a known presence of CKD is taken as a representative example in Supplementary Table S2. In this sample, both REGNN-based frameworks lead the performance in nearly all four criteria with a clear margin, while SSL is even better than the GAE framework. Then we scrutinize the computational methods by comparing their results to the gold standard annotations and mapping them to their original locations in Fig. 4. As a representative CKD sample, the epithelial cell and stroma cell populations are evenly spread, immune and endothelial cells are sparse and spread across the sample. Some competitive methods such as RESEPT (Chang *et al.* 2022), BayesSpace (Zhao *et al.* 2021), SEDR (H. Xu *et al.* 2024), FICT (Teng *et al.* 2022) and CCST (Li *et al.* 2022) only captured large homogeneous cell groups, while missing sparse cell groupings. Other methods like Giotto (Dries *et al.* 2021), and SpaGCN (Hu *et al.* 2021) successfully identify sparse spot clusters but are confused with their cell-type annotations. Both unsupervised and self-supervised versions of REGNN correctly identify the larger sections of epithelial and stroma cells while also identifying some of the sparse spots as endothelial and immune cells. Specifically, REGNN_GAE correctly identifies endothelial cells on the left side of the sample, but it also misses the epithelial cell group on the right side of the sample. REGNN_SSL does better in correctly identifying the epithelial cells on the right portion of the sample, but both frameworks also overrepresent immune and endothelial cells where there were none.

Additionally, we show ARI comparisons on healthy, CKD, and AKI example samples in Supplementary Table S3. Supplementary Fig. S5 displays an AKI sample V10S14-087_XY04_21-0065. We observe similar trends as the CKD sample in Fig. 4, where the competitive methods perform similarly in capturing large cell groupings on this AKI sample, overrepresenting stroma and immune cell populations. REGNN_SSL can identify some of these sparse groupings but misses many of the cell types found on the right side of the sample. Meanwhile, we observe that REGNN_GAE performs well on this AKI sample’s sparse cell groupings, being able to precisely identify areas where immune and stroma populations are located but lack its overall cell population accuracy.

### 3.2 Equivariance and PE are both essential to the expressive power of REGNN

**Figure 5.**
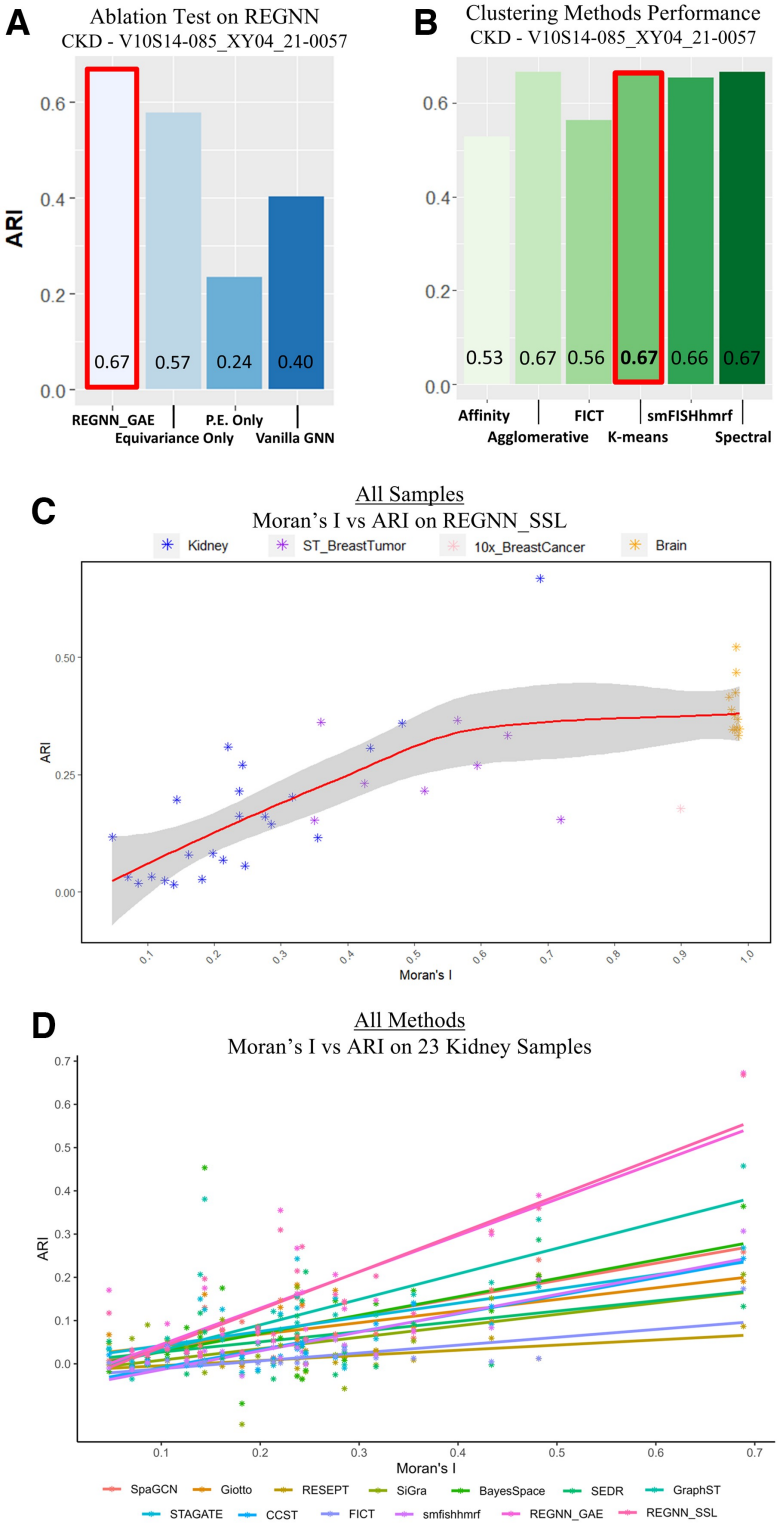
(A) Comparing ablation test results on REGNN_GAE, shown with CKD representative sample V10S14-085_XY04_21-0057. (B) Different Clustering algorithm performance on REGNN_SSL’s graph embeddings on representative CKD sample V10S14-085_XY04_21-0057. (C) REGNN_SSL performance on ARI (*Y*-axis) compared with the sample’s Moran’s I (*X*-axis) on samples used in the study within different heterogeneity. (D) All the comparative methods’ performances on ARI (*Y*-axis) compared with the sample’s Moran’s I (*X*-axis) on the 23 kidney samples used in the study. Both plots (C) and (D) are fit by linear regression to estimate the general trend across the increase in Moran’s I.

We use an ablation test to investigate how the designed equivariance and PE contributed to the performances of REGNN. On REGNN_GAE, we utilize a vanilla GNN, which kept the majority of REGNN model but remove both equivariance in AGG operation and PE in UPDATE operation. Then we only keep equivariance in AGG operation and only keep PE in UPDATE operation. The results of these simplified models are then compared with the REGNN model equipped with both components on three kidney samples, each representing its disease condition. From Fig. 5A, Supplementary Figs 7 and 8, and Supplementary Table S4, we can see that directly utilizing equivariance improves the performance of GNN. While there was slight improvement when only incorporating PE, the combination of both equivariance and PE significantly enhanced the expressive power of REGNN across all three samples. Even in the low ARI reference samples, we observe that the inclusion of equivariance and PE outperforms the vanilla GNN. These results were consistent with the theoretical analysis of the expressive power of the graph deep learning model, where implementing equivariance increases expressive power through enhancing the GNN architecture, and PE increases expressive power by enhancing the topology of the GNN (Sato 2020).

### 3.3 REGNN captures clustering-agnostic representations

Furthermore, we check whether the implemented clustering algorithm, other than the GNN model, plays a significant role in the efficacy of the results. Besides utilizing k-means in REGNN_GAE, five other clustering algorithms including affinity propagation, agglomerative, spectral clustering, FICT, and Hidden-Markov random field are tested on clustering graph embedding from REGNN_SSL from all 23 kidney samples. We show the example of the representative CKD sample V10S14-085_XY04_21-0057 in Fig. 5B and Supplementary Table S5, where the ARI performances of different clustering algorithms are shown to be very close. These results demonstrate the excellence of REGNN’s expressive power, where the learnt embedding clearly represents relations preserved in the learnt presentation, which can be easily detected and captured by various clustering algorithms. As the number of cell classes often overwhelms the ARI score, K-Means is chosen as the clustering algorithm to group the REGNN graph embeddings within the defined number of clusters.

### 3.4 Different graph augmentation strategies do not significantly influence the performance of REGNN representation

Graph augmentation strategies usually play a critical role in self-supervised learning. Each of the four basic graph augmentation strategies, such as node dropping, attribute masking, edge perturbation, and subgraph perturbation augmentation (You *et al.* 2020) is tested with a dropout ratio of 0.1. Part of these results on a CKD, AKI, and reference sample are shown in Supplementary Fig. S7. We can see all four methods performed similarly in terms of resulting scores, with overall medians, means, and standard deviations being very close in the criteria of ARI. Specifically, edge perturbation and node dropping have slightly better results than attribute masking and subgraph perturbation. Edge perturbation and node dropping are further investigated with dropout ratios of 0.05 and 0.2. However, when compared to the same augmentation methods at 0.1 dropout, changing the ratio significantly decreases overall ARI scores. Following these observations, REGNN_SSL is set with edge perturbation with a 0.1 augmentation ratio by default for all testing.

### 3.5 REGNN is designed for analyzing heterogeneous samples

To explore the capacity limitation of REGNN on heterogeneous samples, we further investigate the performance of REGNN on multiple SRT samples within different heterogeneities with manual annotations. Besides 23 kidney samples, these additional samples include: (i) SpatialLIBD(Pardo *et al.* 2022), a widely used dataset which contains 12 samples of the human dorsolateral prefrontal cortex using 10X Visium platform. (ii) A Human Breast Cancer sample (10× Genomics 2019) on 10× Visium platform. (iii) A HER2-positive breast tumor(Andersson 2021) dataset contains eight samples on ST platform. The sample’s heterogeneity is represented by the criteria Moran’s I, the measurement of spatial autocorrelation. Comparing the correlation between ARI and Moran’s I by fitting a linear regression line on all available SRT samples (Fig. 5C), we observe that generally REGNN_SSL shows improved performance with increasing spatial heterogeneity. To focus specifically on our 23 heterogeneous kidney samples, a similar correlation analysis compares ARI scores with other competitive methodologies (Fig. 5D). We observe that both versions of REGNN outperform existing methodologies on samples with spatial heterogeneity of +0.15 and above. This trend is also observed in Supplementary Fig. S10 when using different regression fits.

Additionally, adjusting REGNN’s hyperparameter to 8-dimensional graph embedding improved clustering performance in two-thirds of brain samples (Supplementary Table S6), with cell type predictions more closely resembling benchmark results (Supplementary Figs S11 and S12). These results show that with more fine-tuning on different samples of various spatial autocorrelations, REGNN’s equivariance and positional encoding show potential for broader tissue sample applications.

## 4 Discussion

Graph neural networks are powerful deep learning models on graph data structures, but their inherent expressive power is theoretically limited by their capacities in modeling heterogeneous, symmetric lattices of SRT. We introduced REGNN, an expressive power-enhanced graph deep learning framework designed specifically to model SRT data on highly heterogeneous tissues, such as samples from kidney disease. Compared with several existing computational methods developing on ribbon-like, less heterogeneous brain cortex tissue, REGNN displays its ability to target the more challenging mosaic-like highly heterogeneous tissue by integrating equivariance and positional encoding. The unique strategy of REGNN outperformed these competitors by capturing some intrinsic symmetrical characteristics with better graph-based presentative power of complex heterogeneous patterns in spatial space. Both ablation tests and case studies on multiple kidney samples in various disease conditions further validate this improvement of expressive capacity over a vanilla GNN.

Although REGNN framework achieved some success in kidney studies, there are still limitations in the proposed model. First, the current model is built on the sequencing-based SRT lattice, mainly from the 10× Visium platform. We will continue working on different image-based SRT, such as FISH technologies. Second, current improvements in resolution bring more computational challenges in graph modeling. Compared to the spots in 10× Visium data with thousands of nodes in the modeled graph, other advanced technologies such as MERFISH, 10× Xenium, and NanoString CosMx have hundreds of thousands of nodes in the modeled graph, which brings challenges in the scaling of the graph model. Third, due to its intrinsic, highly heterogeneous nature, accurately inferring the correct architecture on some kidney samples is still very challenging. While performing well on samples with Moran’s I of 0.2, more heterogeneous samples still prove to be a challenge that remains an open problem in the field. Finally, current strategies of histological image integration and graph augmentation in SSL are still far from mature, which may lead to further investigation (see Supplementary Notes S1).

In the future, we will continue improving the REGNN’s expressive power with more advanced positional encoding (Ke *et al.* 2020), and other high-order technologies like Mixhop (Abu-El-Haija *et al.* 2019). Though an attempt was made to incorporate histology image data into the model, other methods may prove more effective and need testing. We are also interested in exploring cutting-edge large language models like LLaMa (Touvron *et al.* 2023) to model the complex relations in the SRT data. Furthermore, we will follow the fast improvements in biologically informed graph SSL (Liu *et al.* 2022), such as node-level and patch-level representation other than currently adopted graph-based representation, and use negative sampling strategies as Barlow twins (Zbontar *et al.* 2021). We will continue fine-tuning REGNN’s model on kidney tissue architecture and other highly heterogeneous mosaic-like tissues such as lymph nodes and colon.

## Supplementary Material

btaf303_Supplementary_Data

## Data Availability

The datasets in this research are publicly available at following resource: (i) 23 10× Visium Kidney samples from CKD, AKI, and Healthy (https://atlas.kpmp.org/repository/), (ii) LIBD human dorsolateral prefrontal cortex, dorsolateral prefrontal cortex 10× Visium data (http://research.libd.org/spatialLIBD/) (iii) Human breast cancer 10× Visium data (https://support.10xgenomics.com/spatial-gene-expression/datasets) (4) HER2-positive breast tumor ST data (https://zenodo.org/records/3957257). REGNN is publicly available at https://doi.org/10.5281/zenodo.15268106 (Mraina 2025).
