# Supplementary material for: Relation equivariant graph neural networks to explore the mosaic-like tissue architecture of kidney diseases on spatially resolved transcriptomics
Source: Bioinformatics. 2025 May 13;41(6):btaf303. doi: 10.1093/bioinformatics/btaf303 (PMC12165735; doi:10.1093/bioinformatics/btaf303)
Supplement: btaf303_Supplementary_Data [file btaf303_supplementary_data.docx]

**Supplementary Notes 1**

**REGNN_SSL implements Histology Image Information using Surrounding Spot Pixel Representations.**

To implement the inclusion of histology image data from the kidney SRT data, we take inspiration from SpaGCN’s^28^ methodology to interpret the relative similarities between spots in the image by using two vertices *u* and *v* to represent the distance between spots. With the distance between the two vertices entailing the similarity between their respective spots, the distance hinges on both the physical proximity of the spots they represent in the tissue slice and the histological characteristics associated with those spots. This is achieved by extending the 2D spatial representation of the tissue slice into a 3D domain that integrates histological data. By using a spot *v*’s pixel coordinates $(x_{pv}, y_{pv})$ in the tissue slice, a 75 x 75-pixel square is centered on the spot coordinates and the mean color value $(r_{v}, g_{v}, b_{v})$ of the pixels within the square is calculated. This approach ensures a more nuanced representation and prevents the dominance of the single pixel color. The weighted sum of the RGB values is taken as Eq (13), where $V_{r}, V_{g}, V_{b}$ represent the variance of $r_{v}, g_{v}, b_{v}$ respectively.

$$\begin{aligned} z_{v}=\frac{(r_{v} \times V_{r}+ g_{v} \times V_{g}+ b_{v} \times V_{b})}{V_{r}+ V_{g}+ V_{b}} \end{aligned}(13)$$

$z_{v}$ is then rescaled according to Eq (14), where $\mu_{v}$ is the mean of $z_{v}$ and the standard deviations of $(x_{v}, y_{v}, z_{v})$ are $(\sigma_{x}, \sigma_{y}, \sigma_{z})$, and $s$ is a scaling factor set to 1, where it can be increased when needing to amplify the weight of the histology information. Spot *v*’s coordinates $(x_{v}, y_{v}, z_{v}^{*})$ are now extended into the 3D space, and lastly the Euclidean distance between two spots u and v is taken as seen in Eq (15).

$$\begin{aligned} z_{v}^{*}=\frac{z_{v}- \mu_{v}}{\sigma_{z}} \times\max(\sigma_{x}, \sigma_{y}) \times s \end{aligned}(14)$$

$$\begin{aligned} d\left( u,v \right)=\sqrt{\left( x_{u}- x_{v} \right)^{2}+ \left( y_{u}- y_{v} \right)^{2}+ \left( z_{u}- z_{v} \right)^{2}} \end{aligned}(15)$$

Using the Euclidean distance, this image information is concatenated with REGNN’s gene expression input data before being run.

**The challenge to integrate Histological Images and REGNN**

Many competitive methodologies have turned to implementing the SRT histology image (H&E) data into their frameworks, such as SiGra^33^ and SpaGCN^28^. To test the capabilities of REGNN_SSL, we implement same idea as SpaGCN^28^ to integrate the kidney SRT samples’ histology image data into REGNN. After isolating the H&E image information from the associated spatial image files, we include that embedding with the sample’s gene expression embedding. A violin plot showing the comparison between the REGNN frameworks can be found in **Supplementary Figure 9**. Compared to the base version of REGNN_SSL, this implementation of H&E data does not show consistent improvement over the existing model. This result shows more elaboration is needed to integrate this image-related information to current model.

**Supplementary Notes 2**

**Benchmarking Processes for SRT Methodologies on 10x Visium Kidney Samples**

**SpaGCN**

We followed the SpaGCN official tutorial with mostly default parameters at https://github.com/jianhuupenn/SpaGCN/blob/master/tutorial/tutorial.ipynb with slight input code modifications to accommodate the extracted 10x Visium data format. Count matrices and spatial coordinates were imported into AnnData objects with an explicit encoding of array and pixel coordinates. The adjacency matrix was calculated using spatial coordinates and the preprocessing pipeline included gene filtering (minimum 3 expressing cells), per-cell normalization, and log transformation of UMI counts. For model optimization, we set p=0.5 and employed the ‘search_l’ function to determine the appropriate l value, followed by resolution parameter optimization to achieve the target number of domains (n=4). The SpaGCN model was then trained for 200 epochs with Louvain initialization and a learning rate of 0.05. Initial clustering results were further refined using a hexagonal neighborhood structure appropriate for Visium data. The resulting spatial domain assignments were evaluated against gold-standard kidney annotations.

**Giotto**

Giotto was run on kidney SRT following the methodology outlined in the Giotto official tutorial at https://drieslab.github.io/Giotto_website/articles/visium_mouse_kidney.html, using a multistep spatial analysis pipeline. A Giotto object was created from the expression matrix, and spatial coordinates were extracted from the base Seurat object. Data underwent quality control and preprocessing following Giotto guidelines, including cell and gene filtering with a minimum of 10 features per cell, features detected in at least 10 cells, normalization using the standard method with a scale factor of 6000, and covariate adjustment for feature count. Highly variable features were identified using covariance-based loess regression. Dimensionality reduction was performed sequentially with PCA. A shared nearest neighbor (sNN) network was constructed using the same dimensions. Different clustering methods were run for spatial domain identification, such as k-means, Leiden, and Louvain clustering. We selected the best-performing Leiden with resolution parameter 0.4 to get close to the target number of clusters needed in the kidney data. The resulting cluster assignments were extracted and evaluated against gold-standard kidney annotations.

**RESEPT**

To benchmark RESEPT, we directly utilized the official tutorial at https://github.com/OSU-BMBL/RESEPT to run 10x Visium Kidney SRT data. Running RESEPT through the command line consisted of performing the data preprocessing, model training, and domain assignment. The kidney 10x Visium data was run through RESEPT, and after training, RESEPT's embedding space was used to predict spatial domains through soft cell type assignment, where each spot received a probability distribution over possible cell types, enabling the identification of spatial regions with mixed cell populations. The resulting spatial domain assignments were evaluated by selecting the highest probability spatial regions against gold standard annotations.

**SiGra**

We followed the official SiGra tutorial with default parameters at https://github.com/QSong-github/SiGra/blob/main/Tutorials/SiGra_train.ipynb. Count matrices and spatial coordinates were loaded into AnnData objects, performing standard preprocessing, including normalization, scaling, and identification of highly variable genes. SiGra's unique graph neural network architecture was then applied, which integrated both gene significance scores and spatial relationships through a multi-layer feature extraction process, ultimately generating a spatially informed latent representation. The SiGra model was trained with default hyperparameters (learning rate=0.001, hidden dimensions=256, epochs=500) with early stopping to prevent overfitting. We note that the necessary change needed for this analysis was to adjust the original workflow to extract the clustering outputs. While the original implementation directly reported ARI values, we extended the script to extract the actual clustering results and subsequently employed a separate Python script to perform comprehensive clustering evaluations.

**BayesSpace**

The methodology outlined in the official BayesSpace DLFPC tutorial at https://www.ezstatconsulting.com/BayesSpace/articles/maynard_DLPFC.html was followed to implement a workflow for spatial domain identification. 10X Visium Kidney data was imported in Seurat data format for the construction of the ‘SingleCellExperiment’ object containing spatial coordinates and gene expression data. Spatial preprocessing was performed using the ‘spatialPreprocess’ function with Visium-specific parameters, followed by dimensionality reduction. Subsequently, spatial clustering was executed with the `spatialCluster` function configured with ‘q=4’ clusters, to match the known number of cell types within the kidney data. The resulting spatial domain assignments were extracted from the ‘SingleCellExperiment’ object and stored as cluster annotations paired with each spot. These derived spatial domains were then used downstream to evaluate the clustering performance of the methodology.

**SEDR**

We run SEDR following the official SEDR clustering tutorial using mostly default parameters at https://sedr.readthedocs.io/en/latest/Tutorial1_Clustering.html with slight input modifications to accommodate our data structures. Count matrices, spatial coordinates, and gold standard annotations were imported and integrated into AnnData objects. The preprocessing workflow involved quality control by filtering genes expressed in fewer than 50 cells or with fewer than 10 counts, followed by total-count normalization to 1 million counts per spot. 2,000 highly variable genes were selected with Seurat v3, and principal component analysis was applied to reduce dimensionality to 159 components. A spatial-molecular graph was constructed using 12 nearest neighbors, capturing both transcriptomic similarities and spatial relationships between spots. The SEDR neural network was initialized in clustering mode and trained using deep embedded clustering for one iteration to learn a latent representation that preserves both expression patterns and spatial organization. The resulting SEDR features were subjected to model-based clustering ‘mclust_R’ with a predetermined number of domains (n=4). The spatial cluster assignments, stored in the 'SEDR' observation field, were evaluated against gold standard kidney annotations.

**GraphST**

We followed GraphST 10x Visium data tutorial with most default parameters at https://deepst-tutorials.readthedocs.io/en/latest/Tutorial%201_10X%20Visium.html. Count matrices, spatial coordinates, and cell type annotations were imported into AnnData objects for each kidney sample. The GraphST model was initialized on a GPU and trained for 600 iterations, using the default hyperparameters to learn spatial representations. We then performed spatial domain identification using the ‘mclust’ algorithm with a predetermined number of 4 clusters, performed directly on the learned latent representations from GraphST's embedding space. The resulting spatial domain assignments were extracted and evaluated against gold standard annotations.

**STAGATE**

STAGATE, specifically STAGATE_pyG, was performed following the STAGATE tutorial at <https://stagate.readthedocs.io/en/latest/T1_DLPFC.html>. Count matrices and spatial coordinates were imported into AnnData objects for each kidney sample. Preprocessing steps included the identification of 3,000 highly variable genes using Seurat v3, followed by normalization to 10,000 counts per spot and log transformation. A crucial step in the STAGATE workflow involved constructing a spatial neighborhood graph with a radius cutoff to define spatial relationships between spots, to integrate spatial information with gene expression profiles. The STAGATE model was then trained using the preprocessed data to generate a low-dimensional embedding space that preserves both gene expression similarity and spatial proximity. Using these STAGATE embeddings, we performed neighborhood graph construction followed by ‘mclust’ clustering with a predefined number of clusters (n=4). The resulting spatial domain assignments were evaluated against gold-standard kidney anatomical annotations.

**CCST**

CCST implementation followed the steps outlined in the CCST GitHub tutorial at https://github.com/xiaoyeye/CCST, with slight input modifications to accommodate our kidney dataset structure. The 'data_generation_ST.py' script was adjusted by editing the get_type() function to enable compatibility with our custom kidney annotations. We processed the kidney samples with non-small cell data type specification (--data_type nsc) and configured the model to identify four distinct spatial domains (--n_clusters 4), consistent with expected kidney architecture. While visualization was generated, differential gene expression analysis was disabled for this evaluation. The resulting spatial domain assignments were extracted and evaluated against gold-standard kidney annotations.

**FICT**

We run FICT following the steps outlined in the FICT and FICT-sample GitHub tutorial at https://github.com/haotianteng/FICT-SAMPLE. To run the pipeline on the extracted 10x Visium kidney data, the files ‘run.py’ and ‘run_seqfish.py’ were adjusted to accommodate this input format change. First, the count matrices and spatial coordinates from kidney Visium data were preprocessed using the standard FICT preprocessing pipeline given in ‘prepare_seqFISH.py’ where the built-in function ‘RealDataLoader’ returned formatted data, which would be compatible with running the main FICT model. No changes were made to the default script, which further processed spatial coordinates, converted them to a standardized scale, found nearest neighbors, and performed dimensionality reduction using PCA.

The reformatted data was then run through cell typing as given in ‘run.py’, which returned the learned distribution of cell type signatures across the tissue. Default model parameters were maintained for the model, excluding cell type initialization, which was set to ‘cell-type=4’ to maintain consistency with other benchmarked methods. The resulting spatial domain assignments were extracted using the FICT API and evaluated against ground truth kidney anatomical annotations using Adjusted Rand Index (ARI) and other metrics. This quantitatively assessed how accurately FICT recapitulated known kidney tissue architecture from spatial transcriptomics data.

FICT was run using both raw counts and embeddings of REGNN. Raw count data was compared to other SRT methods as displayed in Figures 3-4, and 5D. The model was also run using the final embeddings of REGNN to compare to other tested clustering methods, as shown in Figure 5B.

**smFISHHmrf**

Giotto package’s spatialHMRF domains functionality was used to run smFISHhmrf across the 23 distinct kidney samples. Our implementation closely followed the analytical framework established in the Giotto seqFISH+ tutorial, which demonstrates the application of Heterogeneous Markov Random Field (HMRF) for spatial domain identification at <https://rubd.github.io/Giotto_site/articles/mini_seqfish.html#spatial-hmrf-domains>. Leveraging this smFISHhmrf-R implementation of HMRF, count matrices, and spatial coordinates were loaded into the Giotto object and preprocessed following default parameters before defining the spatial grid and spatial network. The resulting spatial domain classifications from the raw data were incorporated into our main analyses, presented in Figures 3-4 and 5D, maintaining consistency by utilizing the same preprocessed input data supplied to REGNN for comparative evaluation. To retrieve clustering results, REGNN’s embeddings were used to create the Giotto object, preprocessing was skipped, and the network and clustering were directly run on final embeddings to get clustering results, as in Figure 5B.

**Supplementary Table 1**. 23 kidney samples from KPMP (Kidney Precision Medicine Project) atlas

| *Samples* | *Type* |
| --- | --- |
| kidney085_XY01_20-0038 | *AKI* |
| kidney085_XY02_20-0040 | *AKI* |
| kidney085_XY03_21-0056 | *AKI* |
| kidney087_XY04_21-0065 | *AKI* |
| kidney086_XY04_21-0066 | *AKI* |
| kidney388_XY02_20-0071 | *AKI* |
| kidney085_XY04_21-0057 | *CKD* |
| kidney086_XY01_21-0055 | *CKD* |
| kidney086_XY02_20-0039 | *CKD* |
| kidney086_XY03_21-0063 | *CKD* |
| kidney087_XY01_21-0061 | *CKD* |
| kidney087_XY02_21-0063 | *CKD* |
| kidney087_XY03_21-0064 | *CKD* |
| kidney388_XY01_21-0068 | *CKD* |
| kidney388_XY03_20-0072 | *CKD* |
| kidney388_XY04_20-0073 | *CKD* |
| kidney017_XY03-13437 | *CKD* |
| kidney102_XY02_IU-21-019-5 | *REF* |
| kidney102_XY03_IU-21-015-2 | *REF* |
| kidney016_XY01_18-0006 | *REF* |
| kidney019_XY02-M32 | *REF* |
| kidney019_XY03-M61 | *REF* |
| kidney019_XY04-F52 | *REF* |

**Supplementary Table 2**. Scoring metrics example on model CKD sample V10S14-085_XY04_21-0057

|  | *ARI* | *NMI* | *RI* | *FMI* |
| --- | --- | --- | --- | --- |
| *SpaGCN* | *0.2586* | *0.3827* | *0.5971* | *0.5706* |
| *Giotto* | *0.1903* | *0.3795* | *0.5561* | *0.5112* |
| *RESEPT* | *0.0866* | *0.1191* | *0.5103* | *0.4755* |
| *SiGra* | *0.2067* | *0.2823* | *0.5732* | *0.5321* |
| *BayesSpace* | *0.3642* | *0.4548* | *0.6671* | *0.6708* |
| *SEDR* | *0.1327* | *0.2857* | *0.5372* | *0.5122* |
| *GraphST* | *0.4574* | *0.7309* | *0.7195* | *0.7309* |
| *STAGATE* | *0.2680* | *0.0871* | *0.5515* | *0.5838* |
| CCST | *0.2680* | *0.3172* | *0.5920* | *0.5688* |
| FICT | *0.2435* | *0.2436* | *0.5494* | *0.5069* |
| smFISHhmrf | *0.1734* | *0.2610* | *0.6325* | *0.6267* |
| *REGNN_GAE* | ***0.6727*** | ***0.5136*** | ***0.8375*** | ***0.8553*** |
| *REGNN_SSL* | *0.6679* | *0.4867* | *0.8352* | *0.8533* |

**Supplementary Table 3**. Comparison of method ARI scores across three main kidney samples

| Samples | V10S14-085_XY04_21-0057 | V10S14-087_XY04_21-0065 | V19S25-019_XY03_M61 |
| --- | --- | --- | --- |
| Status | CKD | AKI | Ref |
| SpaGCN | 0.2586 | 0.1471 | 0.0636 |
| Giotto | 0.1903 | 0.1305 | 0.0554 |
| RESEPT | 0.0866 | 0.0071 | -0.0106 |
| SiGra | 0.2067 | 0.0427 | 0.1770 |
| BayesSpace | 0.3642 | 0.0598 | -0.0285 |
| SEDR | 0.1327 | 0.1119 | 0.1108 |
| GraphST | 0.4574 | 0.0791 | 0.0947 |
| STAGATE | 0.2680 | 0.1157 | **0.2429** |
| CCST | 0.2435 | 0.0177 | ***0.0342*** |
| FICT | 0.1734 | 0.0019 | 0.0018 |
| smFISHhmrf | 0.3069 | 0.0136 | 0.0375 |
| REGNN_GAE | **0.6727** | **0.3550** | 0.1614 |
| REGNN_SSL | 0.6679 | 0.3096 | 0.2148 |

**Supplementary Table 4**. Ablation tests with representative sample V10S14-085_XY04_21-0057

| *Ablation Tests* | *ARI* |
| --- | --- |
| Vanilla GNN (No EGNN or PE) | 0.402 |
| Equivariance only (No PE) | 0.577 |
| PE only (No equivariance) | 0.235 |
| EGNN and PE (REGNN_GAE) | **0.673** |

**Supplementary Table 5**. Performance of different clustering algorithms on REGNN_SSL graph embeddings on representative CKD sample V10S14-085_XY04_21-0057

| Clustering Methods | ARI Score |
| --- | --- |
| FICT | 0.5633 |
| smFISHhmrf | 0.6551 |
| Spectral | 0.6675 |
| Affinity | 0.5284 |
| Agglomerative | 0.6675 |
| K-means | **0.6678** |

**Supplementary Table 6**. REGNN performance on SpatialLIBD, depending on final graph embeddings.

| Brain Sample | Moran's I | 8 Embeddings |
| --- | --- | --- |
| *151507* | 0.9875 | ***0.3470*** |
| *151508* | 0.9856 | 0.3333 |
| *151509* | 0.9822 | 0.4675 |
| *151510* | 0.9768 | ***0.3465*** |
| *151569* | 0.9811 | 0.4248 |
| *151670* | 0.9823 | ***0.5220*** |
| *151671* | 0.9801 | ***0.3760*** |
| *151672* | 0.9715 | 0.4153 |
| *151673* | 0.9803 | ***0.3451*** |
| *151674* | 0.9848 | ***0.3681*** |
| *151675* | 0.9826 | ***0.3512*** |
| *151676* | 0.9752 | ***0.3884*** |

**Supplementary Table 7**. Performance metrics of SRT methods across all 23 10x Visium Kidney Samples

|  | *Mean* | *Standard Deviation* |
| --- | --- | --- |
| *SpaGCN* | *0.0877* | *0.0726* |
| *Giotto* | *0.0787* | *0.0679* |
| *RESEPT* | *0.0189* | *0.0208* |
| *SiGra* | *0.0451* | *0.0845* |
| *BayesSpace* | *0.0866* | *0.1277* |
| *SEDR* | *0.0600* | *0.0825* |
| *GraphST* | *0.1130* | *0.1293* |
| *STAGATE* | *0.0877* | *0.0802* |
| CCST | *0.0489* | *0.0670* |
| FICT | *0.0139* | *0.0358* |
| smFISHhmrf | *0.0468* | *0.0720* |
| *REGNN_GAE* | ***0.1613*** | ***0.1596*** |
| *REGNN_SSL* | *0.1595* | *0.1515* |


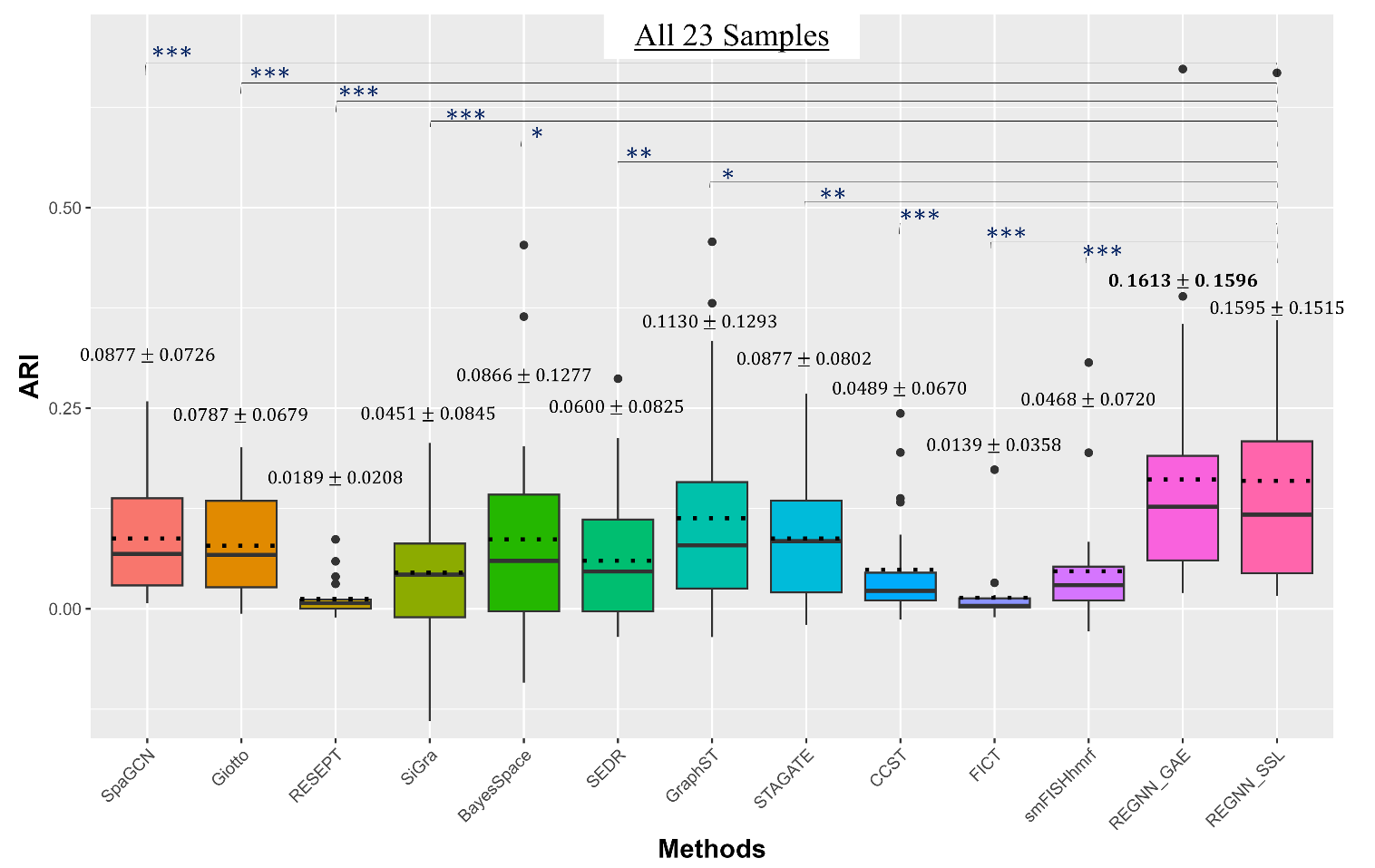


**Supplementary Fig. 1.** Performance comparison on ARI in all samples from KPMP. For each method, the median is indicated by the solid black line and the median is displayed by the dotted line. The Wilcoxon signed rank test was performed to determine the significance of REGNN_SSL’s mean compared with other competitive methods.

*
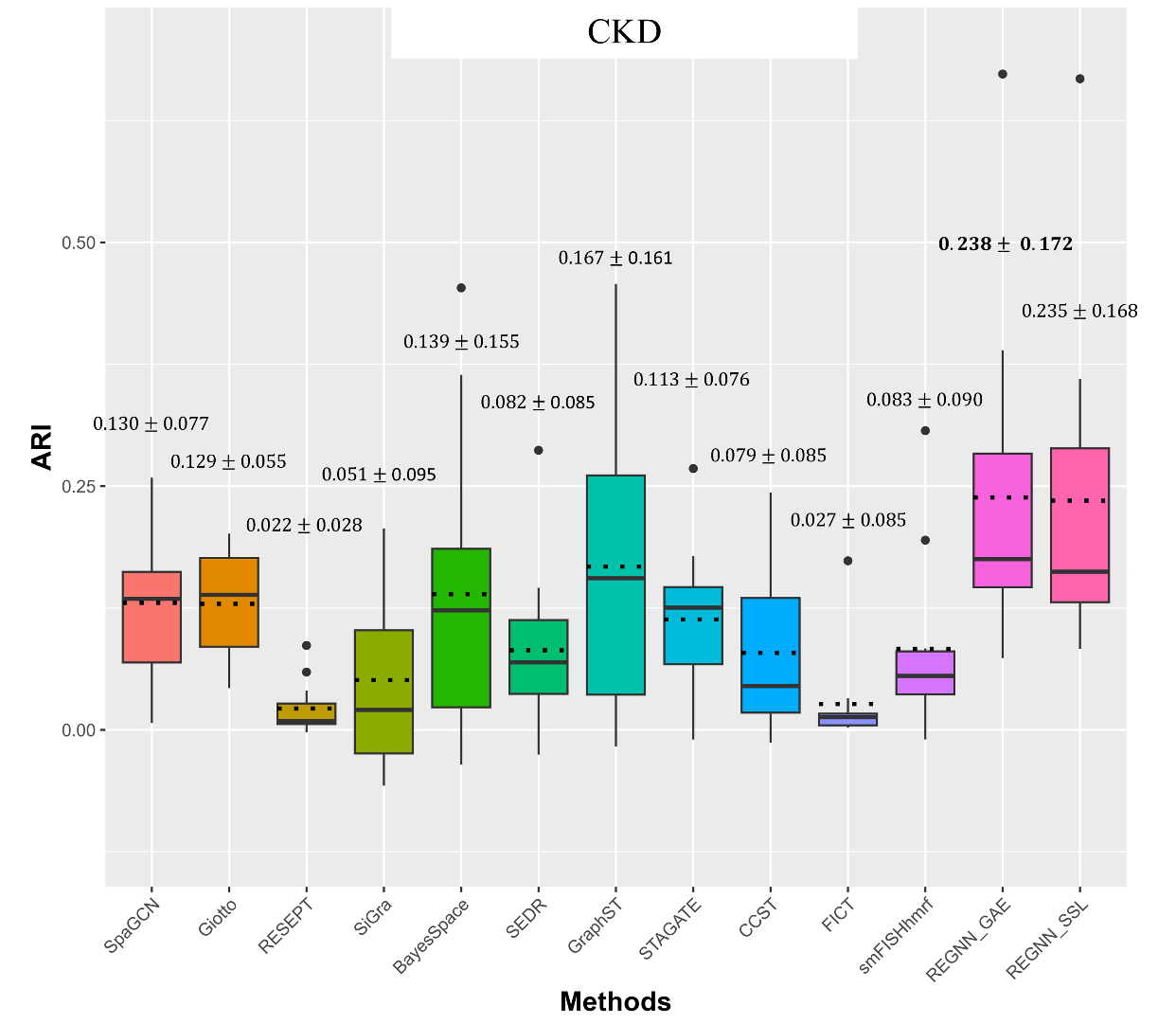
*

**Supplementary Fig. 2**. Performance comparison on ARI in 11 CKD kidney samples from KPMP (Kidney Precision Medicine Project) atlas

*
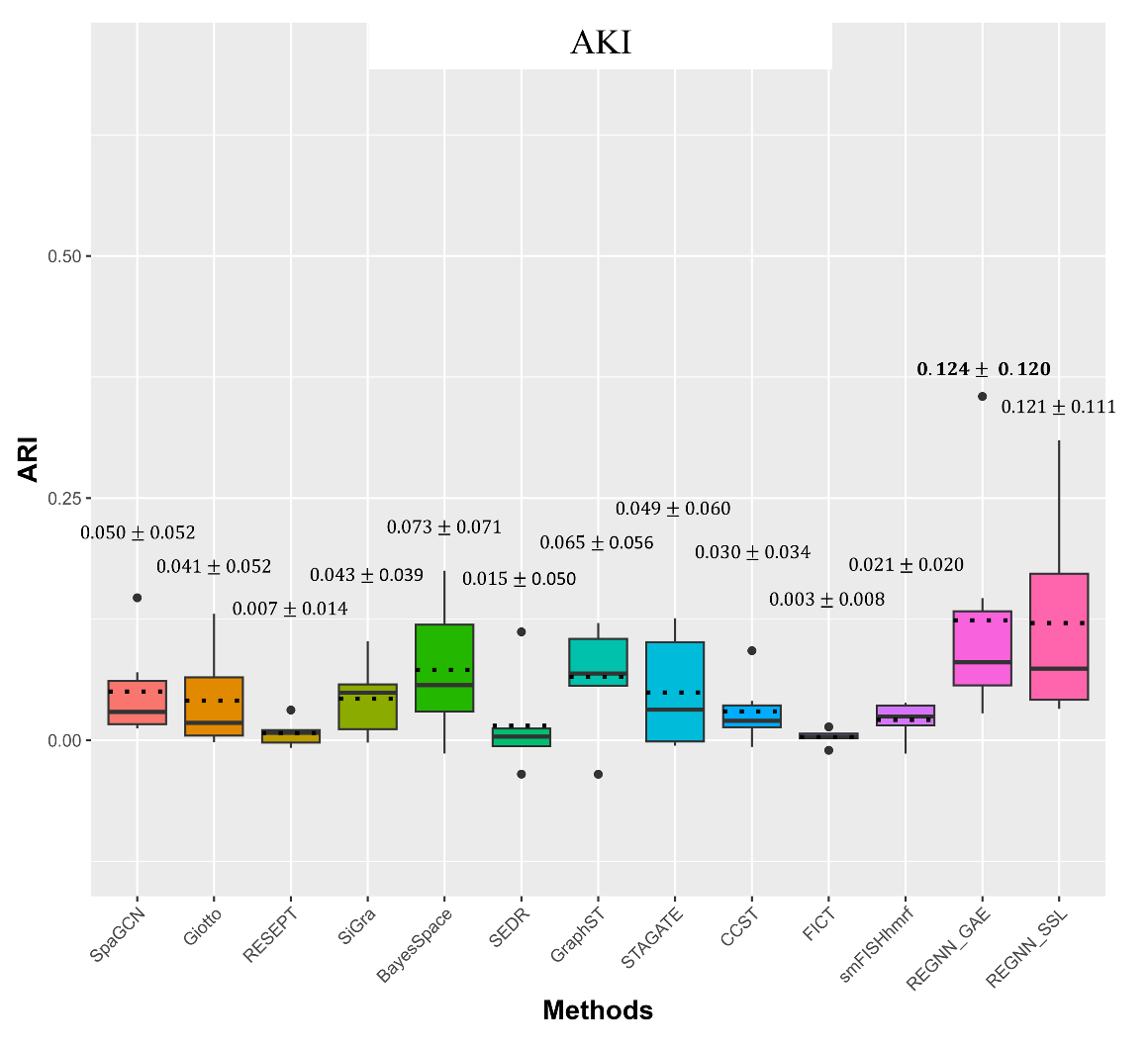
*

**Supplementary Fig. 3**. Performance comparison on ARI in 5 AKI kidney samples from KPMP (Kidney Precision Medicine Project) atlas

*
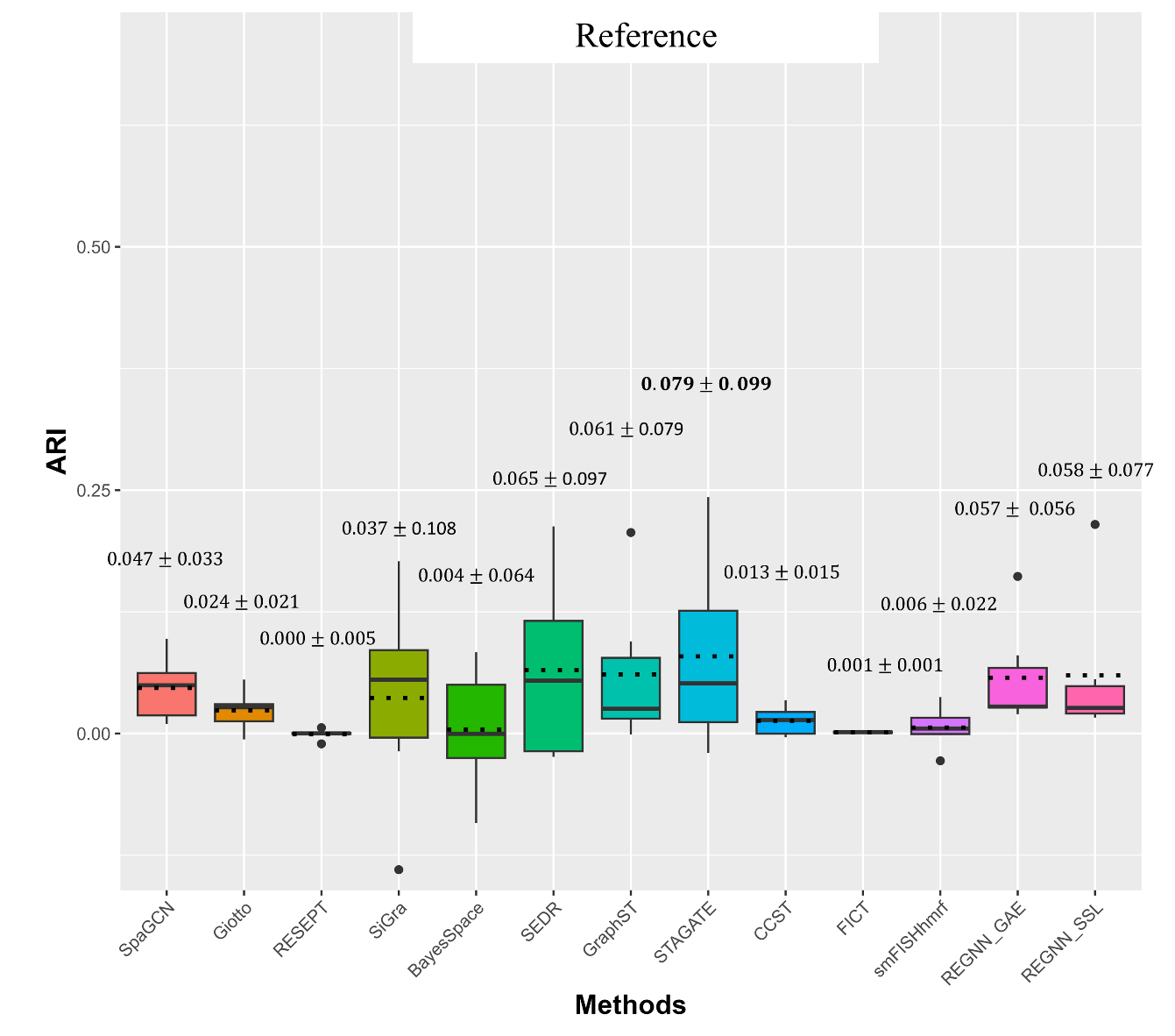
*

**Supplementary Fig. 4**. Performance comparison on ARI in 6 reference kidney samples from KPMP (Kidney Precision Medicine Project) atlas


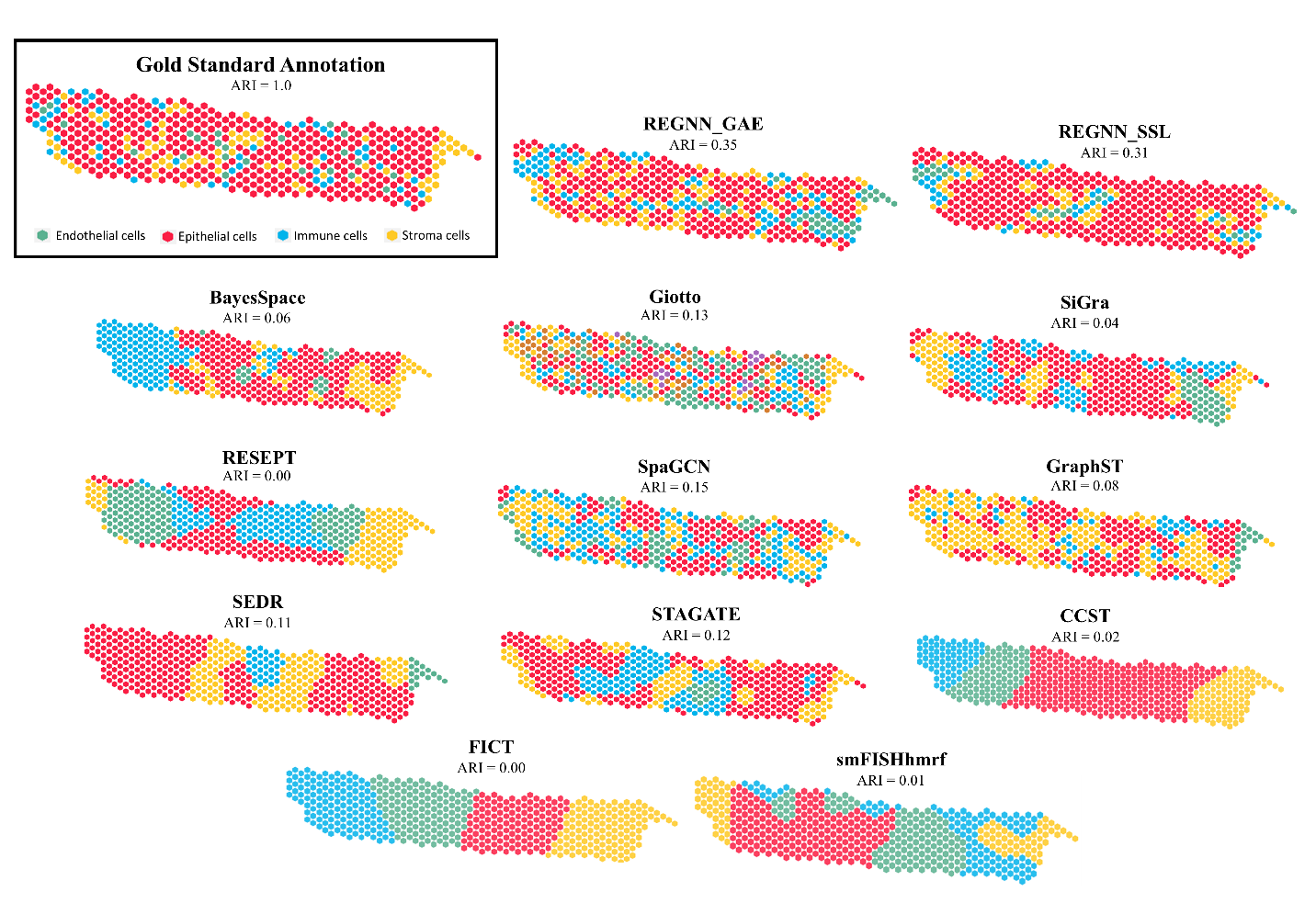


**Supplementary Fig. 5**. Visualization of results from computational methods on AKI sample. The gold standard annotations and calculated results of the computational methods are mapped to original locations of AKI sample V10S14-087_XY04_21-0065.


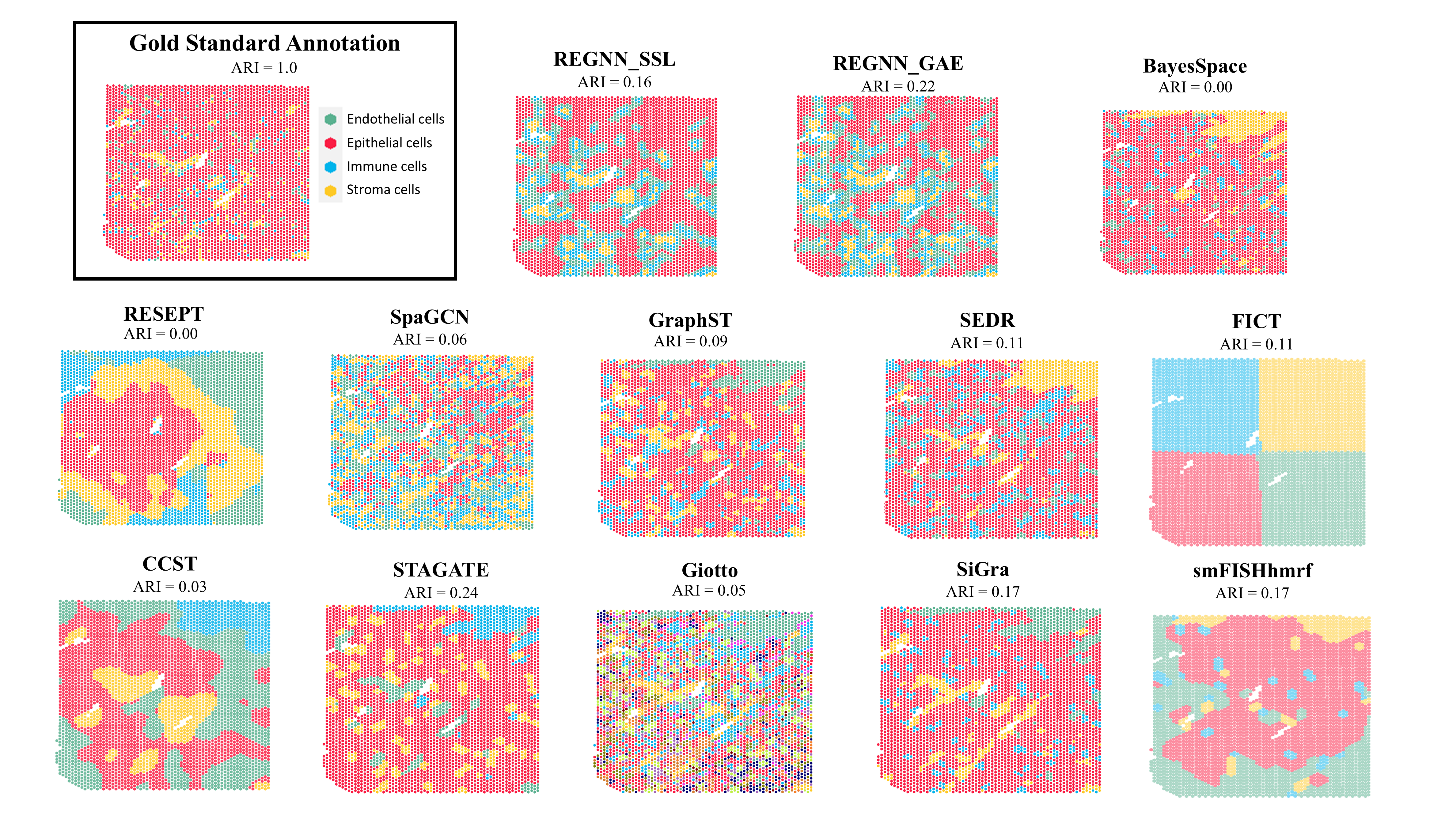


**Supplementary Fig. 6**. Visualization of results from computational methods on Reference sample. The gold standard annotations and calculated results of the computational methods are mapped to original locations of Reference sample V19S25-019_XY03_M61.

*
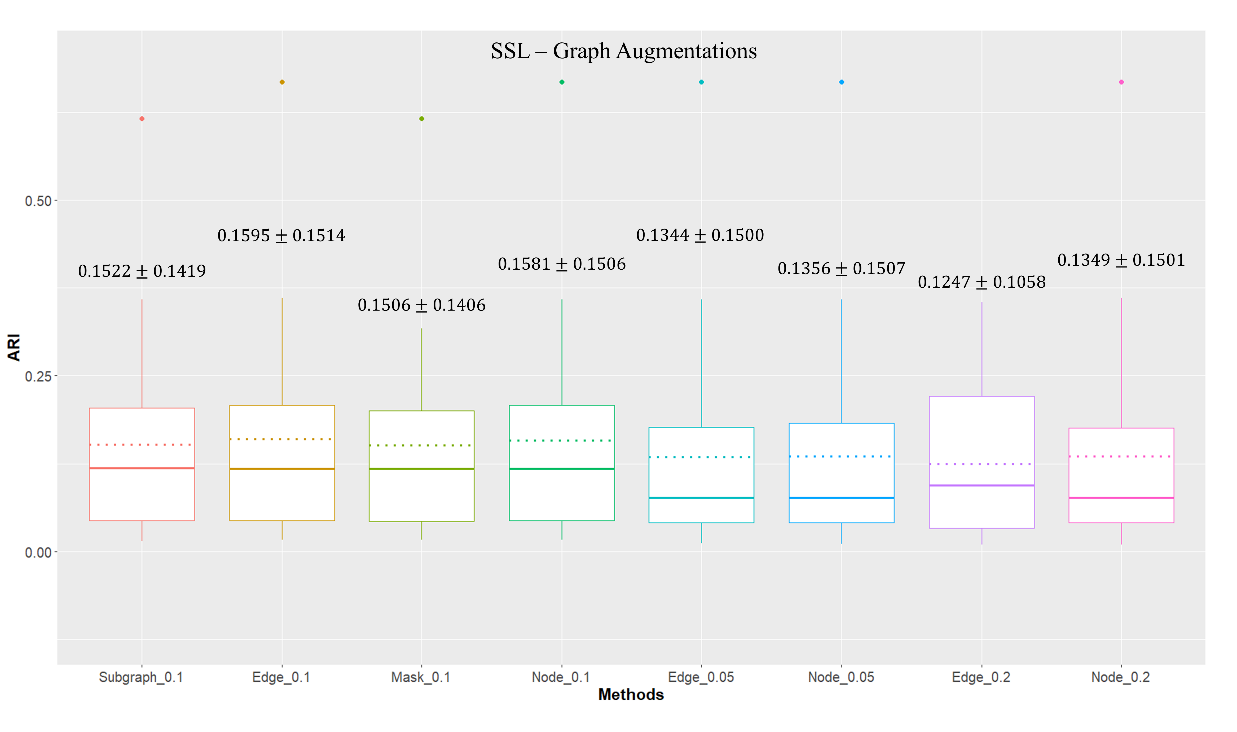
*

**Supplementary Fig. 7**. Performance comparison on ARI in 23 kidney samples from KPMP (Kidney Precision Medicine Project) atlas, comparing different data augmentation methods and dropout ratios for SSL implementation


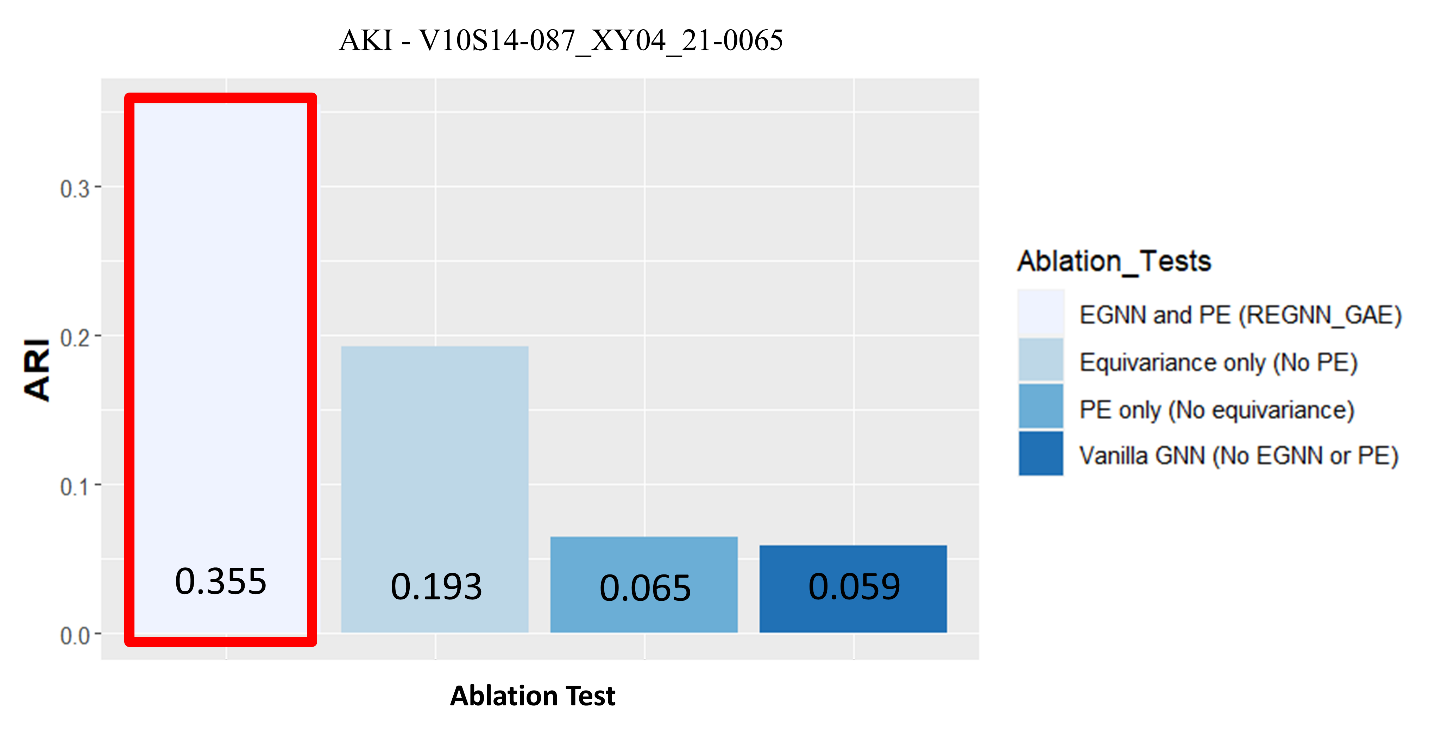


**Supplementary Fig. 8**. Comparing Ablation test results on REGNN_GAE, shown with AKI representative sample V10S14-087_XY04_21-0065


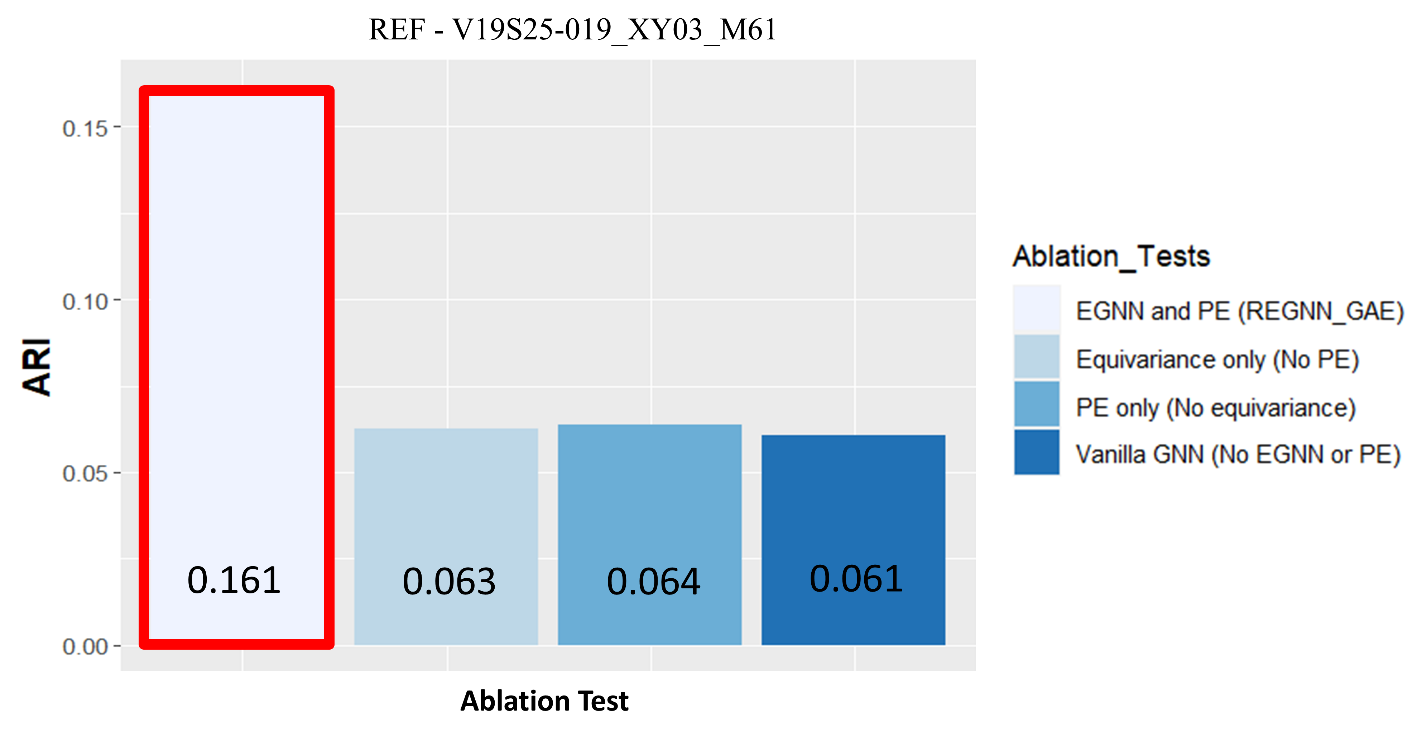


**Supplementary Fig. 9**. Comparing Ablation test results on REGNN_GAE, shown with Reference representative sample V19S25-019_XY03_M61

*
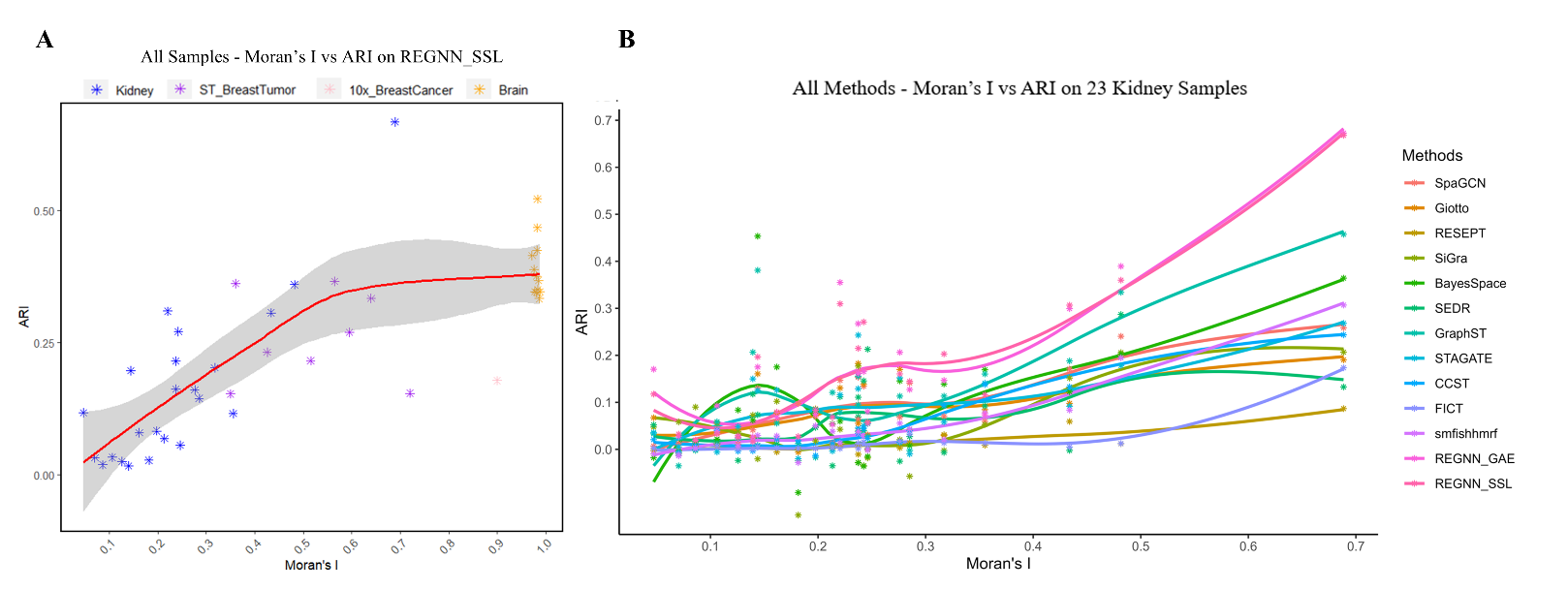
*

**Supplementary Fig. 10**. ARI vs Moran’s I plots which shows the exact points to which the local polynomial regression line is fitted. (A) REGNN_SSL performance on ARI (Y-axis) compared with the sample’s Moran’s I (X-axis) on all samples used in the study showcasing different heterogeneities. (B) All the comparative methods’ performances on ARI (Y-axis) compared with the sample’s Moran’s I (X-axis) on the 23 10x Visium kidney samples

*
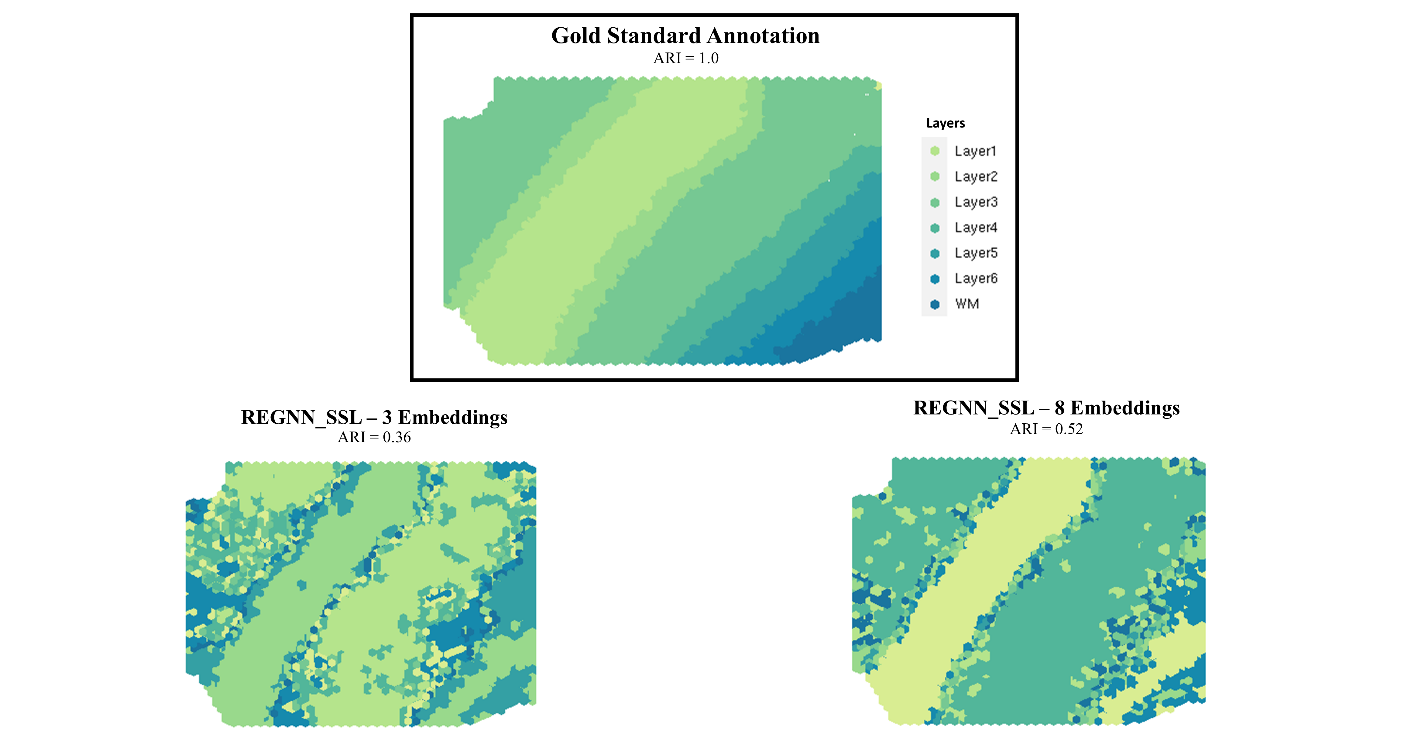
*

**Supplementary Fig. 11**. Visualization of REGNN_SSL results on brain cortex data, comparing the model performance between different tests of final embeddings on SpatialLIBD sample 151509.

*
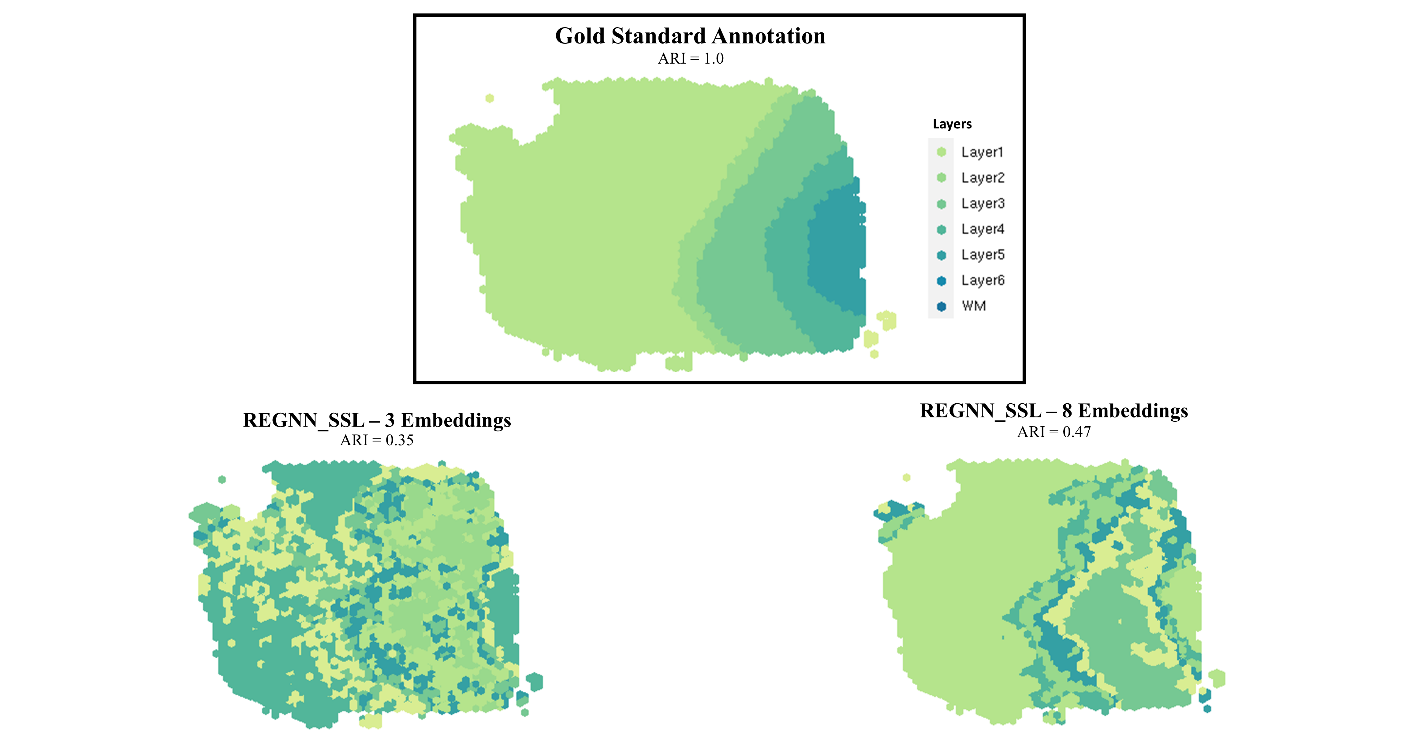
*

**Supplementary Fig. 12**. Visualization of REGNN_SSL results on brain cortex data, comparing the model performance between different tests of final embeddings on SpatialLIBD sample 151670.

*
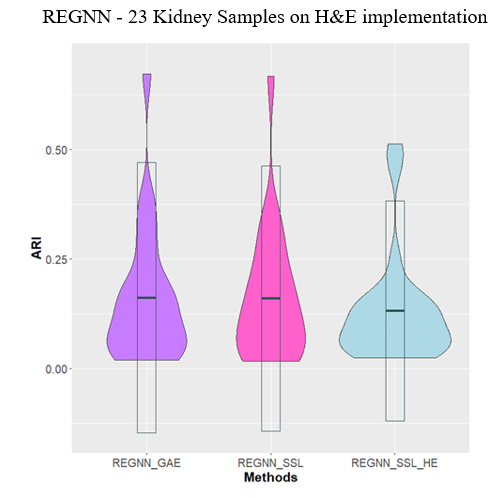
*

**Supplementary Fig. 13**. Comparison of REGNN base frameworks to implementing histology image information into REGNN’s methodology
